# Supplementary material for: Synthetic phage-based approach for sensitive and specific detection of Escherichia coli O157
Source: Commun Biol. 2024 May 6;7:535. doi: 10.1038/s42003-024-06247-w (PMC11074155; doi:10.1038/s42003-024-06247-w)
Supplement: Supplementary file 2 — Supplementary Information [file 42003_2024_6247_MOESM2_ESM.pdf]

Supplementary information

Synthetic phage-based approach for sensitive and specific detection of *Escherichia coli* O157

Azumi Tamura[1,2], Aa Haeruman Azam[1], Tomohiro Nakamura[1], Kenichi Lee[3], Sunao Iyoda[3], Kohei Kondo[1], Shinjiro Ojima[1], Kotaro Chihara[1], Wakana Yamashita[1,4], Longzhu Cui[5], Yukihiro Akeda[3], Koichi Watashi[1], Yoshimasa Takahashi[1,4], Hiroshi Yotsuyanagi[2], Kotaro Kiga[1,5]

[1] Research Center for Drug and Vaccine Development, National Institute of Infectious Diseases, Shinjuku-ku, Tokyo, Japan
[2] Division of Infectious Diseases, Advanced Clinical Research Center, The Institute of Medical Science, The University of Tokyo, Minato-ku, Tokyo, Japan
[3] Department of Bacteriology I, National Institute of Infectious Diseases, Shinjuku-ku, Tokyo, Japan
[4] Department of Life Science and Medical Bioscience, Waseda University, Shinjuku-ku, Tokyo, Japan
[5] Division of Bacteriology, Department of Infection and Immunity, School of Medicine, Jichi Medical University, Shimotsuke-shi, Tochigi, Japan

Corresponding author: Kotaro Kiga
Email: k-kiga@niid.go.jp

Supplementary Fig. 1: Analysis of O157_vB-resistant mutants.

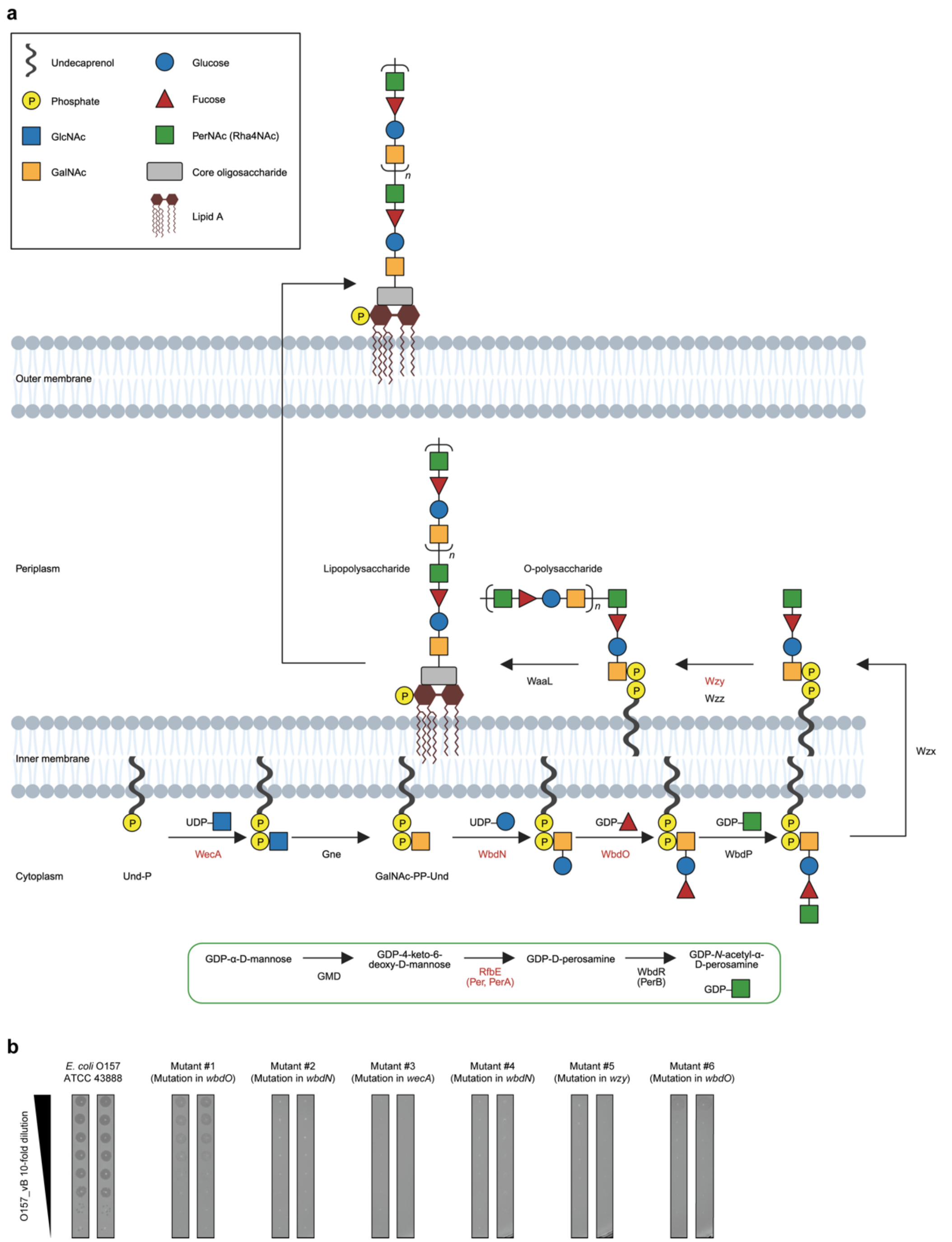

a Proposed biosynthesis pathway for *E. coli* O157 O antigen. Figures in Li *et al*.[1] and Gao *et al*.[2] were modified. The pathway for biosynthesis of GDP-*N*-acetyl-α-D-perosamine catalyzed by RfbE (Per, PerA)[3] is also included. Enzyme names in red represent RfbE or gene products in which mutations were observed in O157_vB-resistant mutants. This figure was created using BioRender.com (accessed August 2023). **b** Lytic activity of O157_vB against *E. coli* O157 (ATCC 43888) mutants. Spot tests were performed twice for each mutant.

Supplementary Fig. 2: Uncropped image of spot assays.

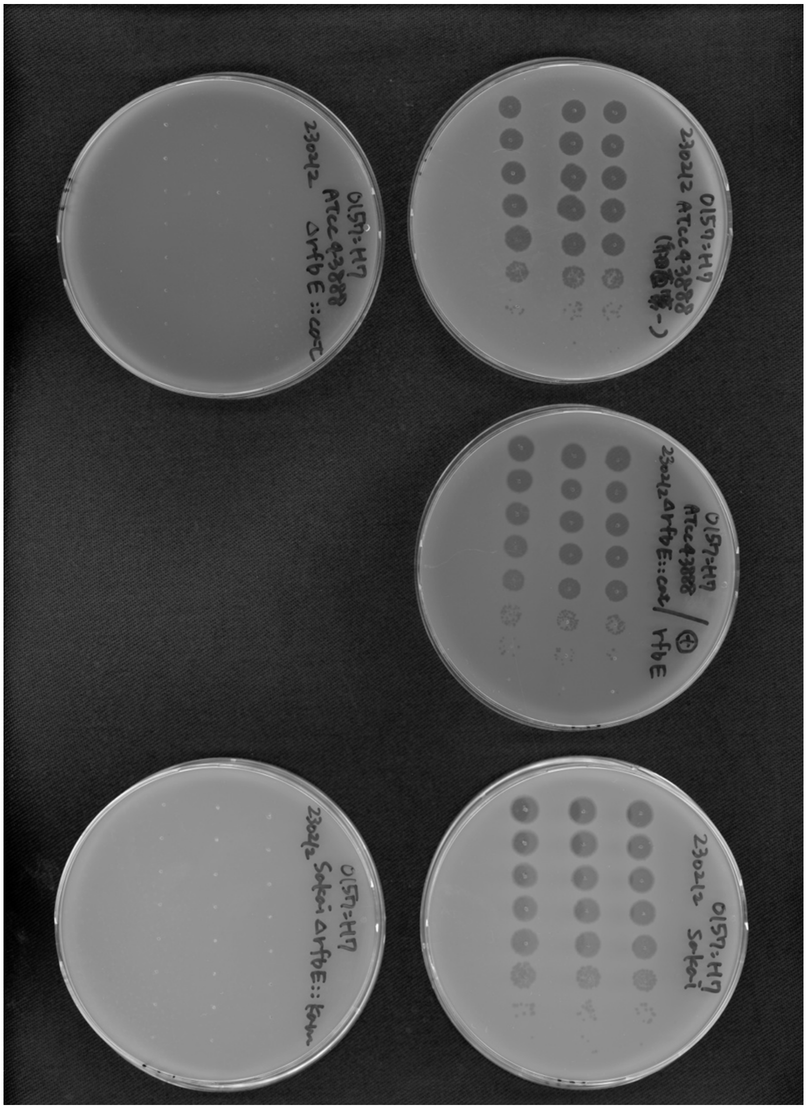

Uncropped images of spot assays of O157_vB against *rfbE*-deficient *E. coli* O157 strains shown (cropped) in Fig. 2b.

Supplementary Fig. 3: Optimization of phage synthesis.

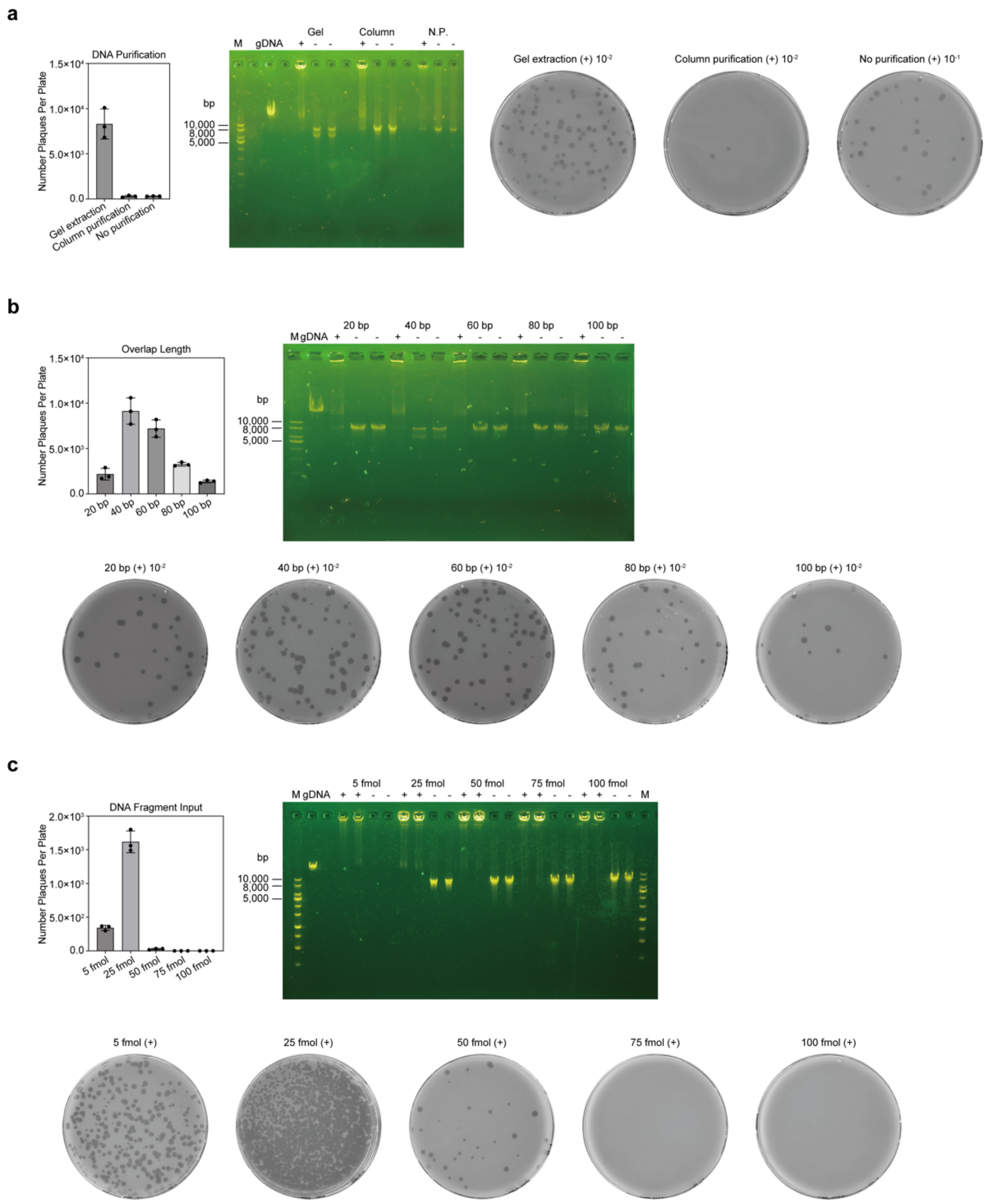

To enhance efficiency of in vitro phage synthesis, several conditions were considered using T7 phages. **a** Consideration of DNA purification methods. PCR fragments were treated using gel purification, column-based DNA purification, or no purification. **b** Examination of length of overlapping regions between adjacent fragments. Overlapping regions were designed to be 20, 40, 60, 80, or 100 bp in length, and five sets of PCR fragments were prepared. **c** Consideration of the amount of DNA required for assembly. Purified DNA fragments at equimolar concentrations (5, 25, 50, 75, or 100 fmol) were reacted with assembly enzymes. Three replicates were performed for each condition. The number of plaques per plate (on the left of each panel) was calculated using the dilution ratio of assembled samples before electroporation. Results are presented as each replicate and the mean with standard deviation (SD). Samples (with or without assembly enzymes) and genomic DNA (70–200 ng) were loaded onto agarose gel. The contrast and sharpness of the plate images on the right of each panel were altered identically. M: 1 kb XL-DNA Ladder (Pharma Foods International Co., Ltd., Kyoto, Japan).

Supplementary Fig. 4: Rebooting of O157_vB from DNA fragments.

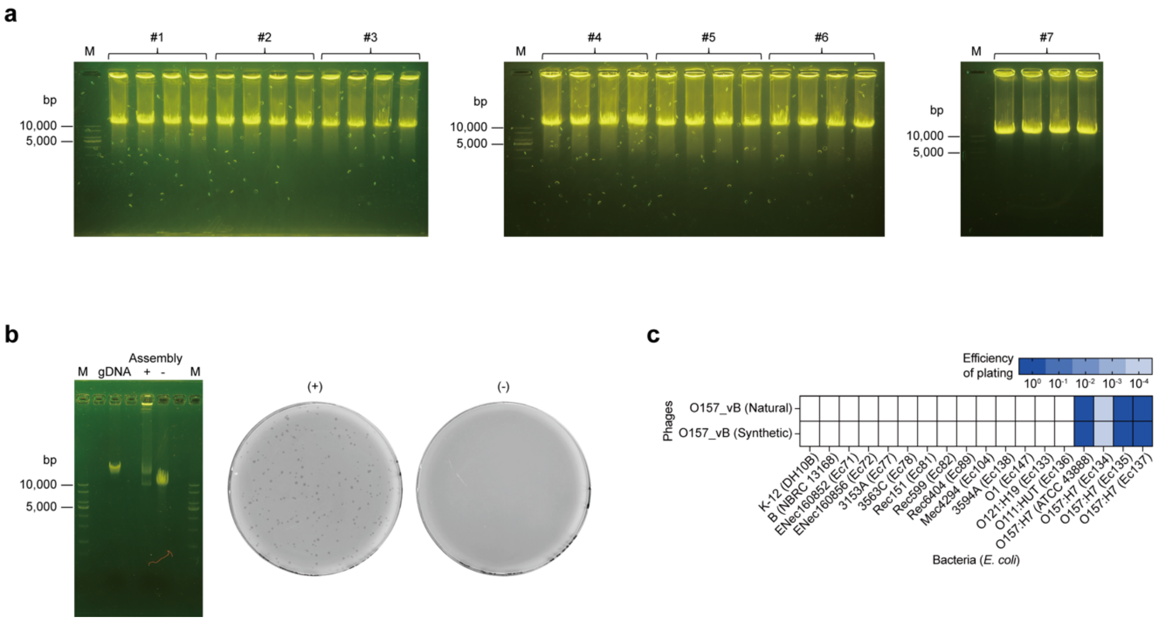

a PCR fragments used for synthesis of O157_vB phage. Seven 10 kb fragments were amplified. **b** Gel (left) and plate (right) images of assembled and unassembled samples. A sample without assembly enzymes was used as the negative control. After the genome assembly step, a portion of sample and genomic DNA (250 ng) was loaded onto agarose gel. The contrast and sharpness of the plate images were altered identically. M: 1 kb XL-DNA Ladder (Pharma Foods International Co., Ltd., Kyoto, Japan). **c** Evaluation of lytic activities of natural and synthetic O157_vB phages.

Supplementary Fig. 5: Rebooting of HiBiT-tagged O157_vB phages from DNA fragments.

a

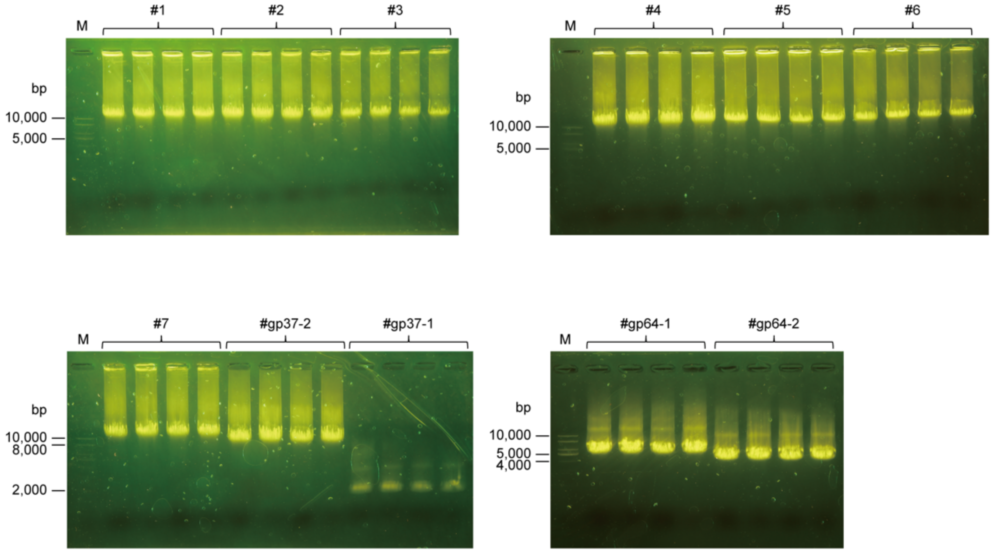

b

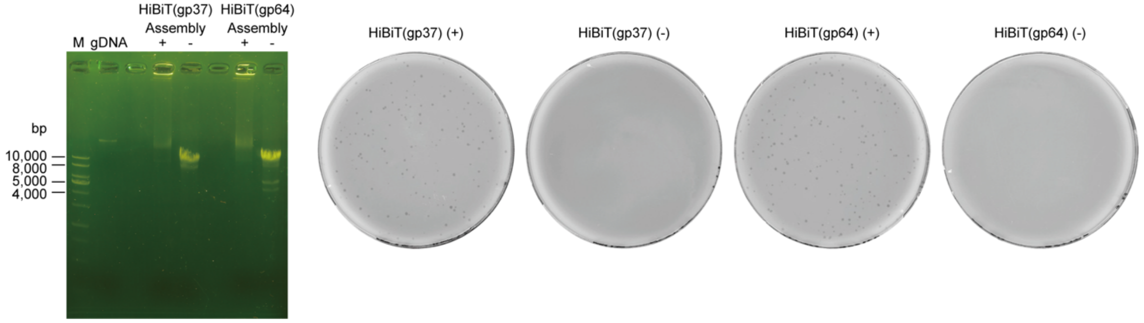

a PCR fragments used for synthesis of HiBiT-tagged O157_vB phages. Seven common fragments and four fragments containing HiBiT sequence were amplified. **b** Gel and plate images of assembled and unassembled samples. Samples without assembly enzymes were used as negative controls. After the genome assembly step, a portion of sample and genomic DNA (100 ng) were loaded onto agarose gel. Contrast and sharpness of plate images were altered identically. M: 1 kb XL-DNA Ladder (Pharma Foods International Co., Ltd., Kyoto, Japan).

Supplementary Fig. 6: Lytic activity of synthetic O157_vB phages against various *E. coli* strains.

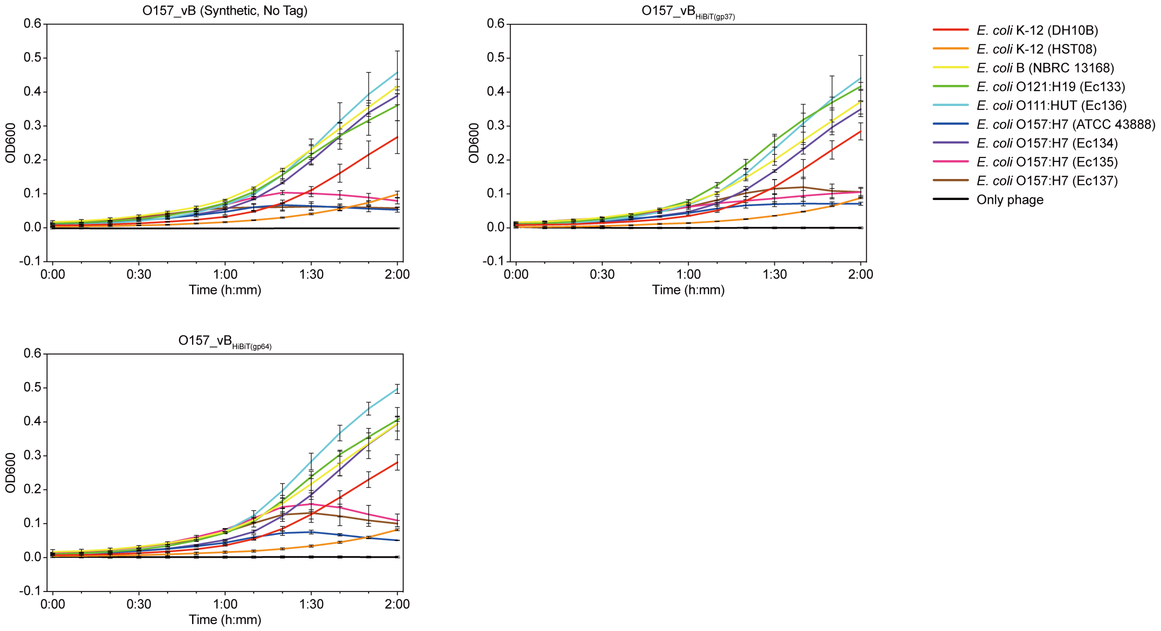

Phages and bacteria were mixed at multiplicity of infection of 1 and incubated at 37 °C for 2 h with shaking. Optical density (OD600) was measured at 10-min intervals. Three technical replicates were used for each experiment. Results are shown as connecting lines with error bars (mean with SD).

Supplementary Fig. 7: Rebooting of 20 O157_vB phages with HiBiT tags.

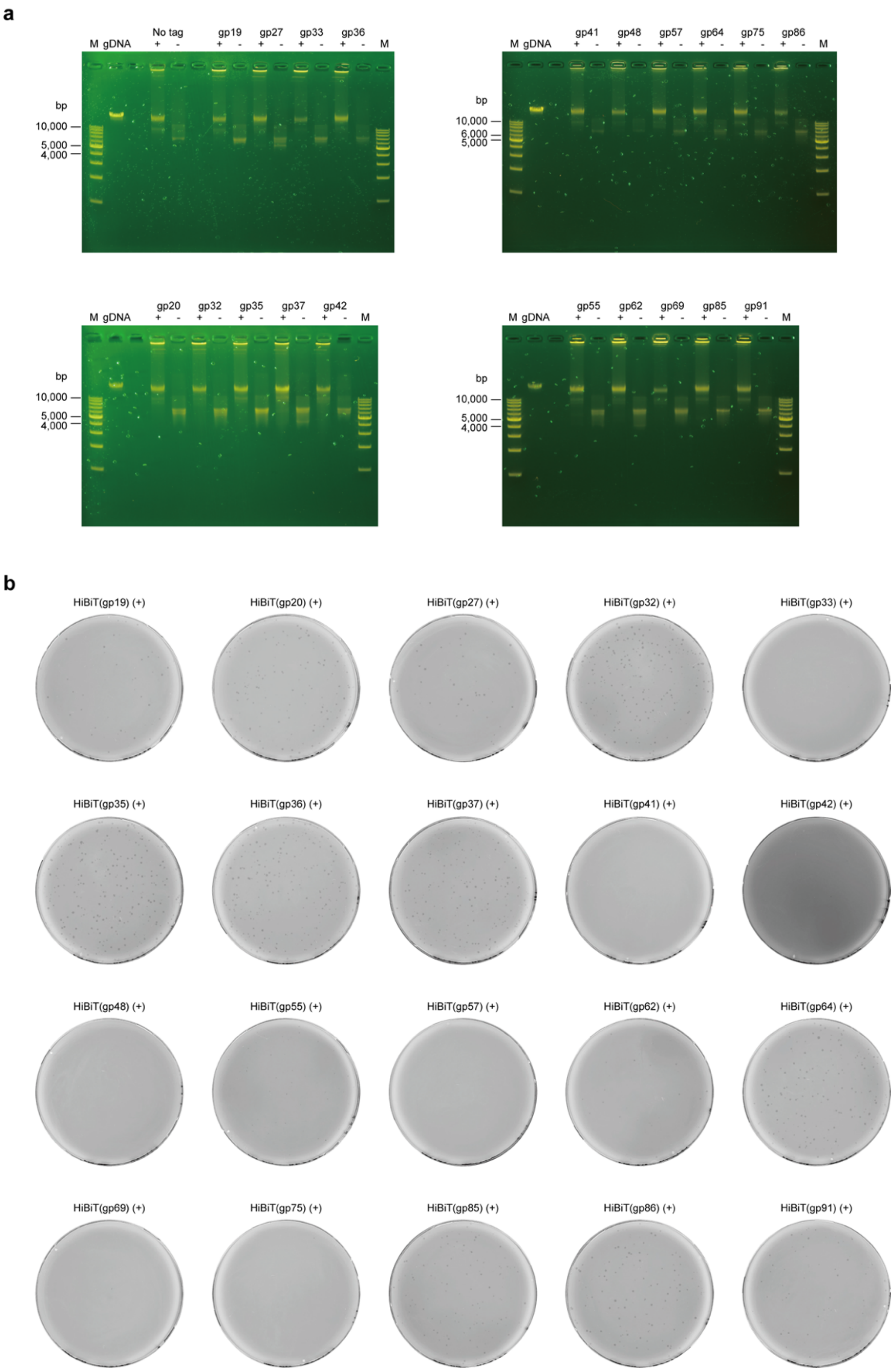

a Electrophoresis of assembled samples. Samples (with or without assembly enzymes) and genomic DNA (90 ng) were loaded onto agarose gels. M: 1 kb DNA Ladder (Takara Bio Inc., Shiga, Japan). **b** Plate images of 20 tagged phages. Contrast and sharpness of the plate images were altered identically.

Supplementary Fig. 8: Working curves for *E. coli* O157 detection with HiBiT-tagged O157_vB.

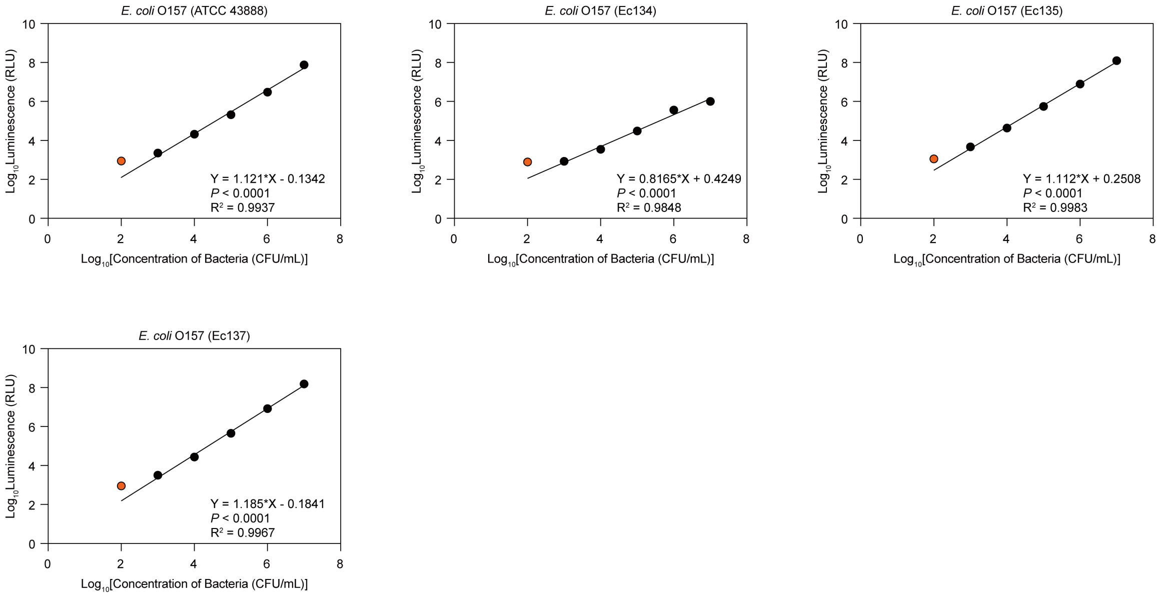

Working curves were generated using simple linear regression based on luminescence data from 2-hour co-incubation of O157_vB$_{HiBiT(gp27)}$ with ten-fold serially diluted *E. coli* O157 at 10^3–10^7 CFU/mL. Luminescence levels are displayed as points representing the average of three replicates. Points in red represent data from 10^2 CFU/mL *E. coli* O157 that were not used in working curves.

Supplementary Table 1: SNP analysis of phage-resistant *E. coli* O157 mutants.
Supplementary Table 2: Statistical analysis of luminescence levels among tagged phages.
Supplementary Table 3: Statistical analysis of luminescence levels at different *E. coli* O157 concentrations.
Supplementary Table 4: Statistical analysis of luminescence levels at different *E. coli* O157 pre-incubation times.
Supplementary Table 5: Phage defense systems searched in *E. coli* O157 clinical isolates.
Supplementary Table 6: Comparison of *E. coli* O157 detection methods.
Supplementary Table 7: Phage and bacterial strains used in this study.
Supplementary Table 8: PCR primers used in this study.
Supplementary Table 9: PCR conditions for amplification of phage DNA fragments.
Supplementary Table 10: PCR conditions for amplification of HiBiT sequence.
Supplementary Table 11: Whole genome re-sequencing of phage vB_Eco4M-7.

Supplementary Table 1: SNP analysis of phage-resistant *E. coli* O157 mutants.

Mutant no.	Variant type	Nucleotide(s) in reference	Alternate nucleotide(s) supported by reads	Frequency counts	Nucleotide position	Residue position	Annotated consequence of variant	Gene	Product
1	SNP	A	C	C:246 A:0	398/747	133/248	missense_variant Val133Gly	*wbdO*	glycosyltransferase (locus tag: FNZ21_14185)
2	SNP	G	T	T:193 G:0	248/783	83/260	missense_variant Ala83Asp	*wbdN*	glycosyltransferase (FNZ21_14175)
3	SNP	G	A	A:212 G:0	793/1104	265/367	missense_variant Arg265Cys	*wecA*	UDP-N-acetylglucosamine--undecaprenyl-phosphate N-acetylglucosaminephosphotransferase (FNZ21_07475)
4	SNP	A	C	C:179 A:0	587/783	196/260	missense_variant Leu196Arg	*wbdN*	glycosyltransferase (FNZ21_14175)
5	INS	G	GC	GC:195 G:0	316/1185	106/394	frameshift_variant Ala106fs	*wzy*	O157 family O-antigen polymerase (FNZ21_14180)
6	SNP	A	C	C:215 A:0	398/747	133/248	missense_variant Val133Gly	*wbdO*	glycosyltransferase (FNZ21_14185)

SNP: single nucleotide polymorphism

INS: insertion

Supplementary Table 2: Statistical analysis of luminescence levels among tagged phages.

One-way ANOVA with Brown–Forsythe and Welch tests	Dunnett's T3 multiple comparisons test	Is gp27 higher?	Mean difference	95.00% CI of difference	Below threshold (0.05)?	Summary	Adjusted *P*-value
E. coli O157 (ATCC 43888)	gp27 vs. No tag	Yes	59641333	32938601 to 86344064	Yes	*	0.0103
	gp27 vs. gp19	Yes	35940000	9072936 to 62807064	Yes	*	0.0283
	gp27 vs. gp20	Yes	56845667	30093509 to 83597824	Yes	*	0.0114
	gp27 vs. gp32	Yes	58750133	32047386 to 85452880	Yes	*	0.0106
	gp27 vs. gp35	Yes	56526667	29822863 to 83230471	Yes	*	0.0114
	gp27 vs. gp36	Yes	48796667	22082868 to 75510466	Yes	*	0.0153
	gp27 vs. gp37	Yes	58678433	31975614 to 85381253	Yes	*	0.0106
	gp27 vs. gp55	Yes	56042000	29312544 to 82771456	Yes	*	0.0117
	gp27 vs. gp64	Yes	43096667	16217557 to 69975776	Yes	*	0.0198
	gp27 vs. gp75	Yes	39893333	12867548 to 66919119	Yes	*	0.0233
	gp27 vs. gp85	Yes	59476400	32773574 to 86179226	Yes	*	0.0103
	gp27 vs. gp86	Yes	56678333	29970880 to 83385787	Yes	*	0.0114
	gp27 vs. gp91	Yes	42846667	16081851 to 69611482	Yes	*	0.0199
E. coli O157 (Ec134)	gp27 vs. No tag	Yes	735079	665290 to 804868	Yes	***	0.0005
	gp27 vs. gp19	No	-172867	-281701 to -64033	Yes	*	0.0136
	gp27 vs. gp20	Yes	390767	318484 to 463049	Yes	**	0.0018
	gp27 vs. gp32	Yes	631767	561969 to 701564	Yes	***	0.0006
	gp27 vs. gp35	Yes	350967	298100 to 403833	Yes	***	0.0002
	gp27 vs. gp36	Yes	547233	494554 to 599913	Yes	****	<0.0001
	gp27 vs. gp37	Yes	707980	638186 to 777774	Yes	***	0.0005
	gp27 vs. gp55	Yes	700730	630082 to 771378	Yes	***	0.0005
	gp27 vs. gp64	Yes	499633	429477 to 569790	Yes	**	0.001
	gp27 vs. gp75	Yes	344400	296086 to 392714	Yes	****	<0.0001
	gp27 vs. gp85	Yes	731937	662147 to 801726	Yes	***	0.0005
	gp27 vs. gp86	No	-269633	-320860 to -218407	Yes	***	0.0004
	gp27 vs. gp91	Yes	323867	267714 to 380020	Yes	***	0.0003
E. coli O157 (Ec135)	gp27 vs. No tag	Yes	96721024	87637927 to 105804122	Yes	***	0.0005
	gp27 vs. gp19	Yes	76876667	67593284 to 86160049	Yes	***	0.0008
	gp27 vs. gp20	Yes	93085667	83984654 to 102186680	Yes	***	0.0005
	gp27 vs. gp32	Yes	94440000	85356836 to 103523164	Yes	***	0.0005
	gp27 vs. gp35	Yes	94316333	85043771 to 103588896	Yes	***	0.0005
	gp27 vs. gp36	Yes	81800000	75087758 to 88512242	Yes	****	<0.0001
	gp27 vs. gp37	Yes	95353667	86243841 to 104463492	Yes	***	0.0005
	gp27 vs. gp55	Yes	91279667	82187379 to 100371954	Yes	***	0.0005
	gp27 vs. gp64	Yes	57976667	51398557 to 64554776	Yes	****	<0.0001
	gp27 vs. gp75	Yes	54456667	48115510 to 60797824	Yes	****	<0.0001
	gp27 vs. gp85	Yes	96539300	87456181 to 105622419	Yes	***	0.0005
	gp27 vs. gp86	Yes	89643000	80536415 to 98749585	Yes	***	0.0005
	gp27 vs. gp91	Yes	72683333	66152036 to 79214631	Yes	****	<0.0001
E. coli O157 (Ec137)	gp27 vs. No tag	Yes	76491371	64812120 to 88170622	Yes	**	0.0012
	gp27 vs. gp19	Yes	58286667	46349148 to 70224185	Yes	**	0.0022
	gp27 vs. gp20	Yes	73465333	61730130 to 85200537	Yes	**	0.0013
	gp27 vs. gp32	Yes	74519333	62838307 to 86200360	Yes	**	0.0013
	gp27 vs. gp35	Yes	75016000	63330797 to 86701203	Yes	**	0.0013
	gp27 vs. gp36	Yes	64123333	52378240 to 75868427	Yes	**	0.0017
	gp27 vs. gp37	Yes	75548200	63867937 to 87228463	Yes	**	0.0012
	gp27 vs. gp55	Yes	72316000	60576250 to 84055750	Yes	**	0.0014
	gp27 vs. gp64	Yes	45823333	36686500 to 54960166	Yes	***	0.0005
	gp27 vs. gp75	Yes	38153333	26760693 to 49545974	Yes	***	0.0005
	gp27 vs. gp85	Yes	76311000	64630686 to 87991314	Yes	**	0.0012
	gp27 vs. gp86	Yes	69762333	58064699 to 81459968	Yes	**	0.0015
	gp27 vs. gp91	Yes	54936667	46388036 to 63485298	Yes	***	0.0002
Only phage	gp27 vs. No tag	Yes	1984	1767 to 2200	Yes	****	<0.0001
	gp27 vs. gp19	Yes	1504	1296 to 1712	Yes	***	0.0002
	gp27 vs. gp20	Yes	1784	1570 to 1997	Yes	****	<0.0001
	gp27 vs. gp32	Yes	1822	1538 to 2107	Yes	**	0.0013
	gp27 vs. gp35	Yes	1860	1576 to 2144	Yes	**	0.0012
	gp27 vs. gp36	No	-2935	-3162 to -2709	Yes	****	<0.0001
	gp27 vs. gp37	Yes	1488	1264 to 1712	Yes	***	0.0002
	gp27 vs. gp55	Yes	749	508.4 to 989.6	Yes	***	0.0007
	gp27 vs. gp64	Yes	633	345.4 to 920.6	Yes	*	0.0106
	gp27 vs. gp75	No	-1914	-2308 to -1520	Yes	***	0.0005
	gp27 vs. gp85	Yes	1914	1628 to 2200	Yes	**	0.0012
	gp27 vs. gp86	Yes	1740	1455 to 2024	Yes	**	0.0014
	gp27 vs. gp91	Yes	1514	1297 to 1732	Yes	***	0.0002

In the summary column, significance is indicated as *$P \leq 0.05$, **$P \leq 0.01$, ***$P \leq 0.001$, or ****$P \leq 0.0001$.

Supplementary Table 3: Statistical analysis of luminescence levels at different *E. coli* O157 concentrations.

One-way ANOVA with Brown–Forsythe and Welch tests	Dunnett's T3 multiple comparisons test	Mean difference	95.00% CI of difference	Below threshold (0.05)?	Summary	Adjusted P-value
E. coli O157 (ATCC 43888)	0 vs. 10^7	-76064702	-81723040 to -70406365	Yes	***	0.0003
	0 vs. 10^6	-3020702	-3630840 to -2410565	Yes	**	0.0021
	0 vs. 10^5	-209302	-221442 to -197163	Yes	***	0.0002
	0 vs. 10^4	-20349	-21832 to -18866	Yes	***	0.0003
	0 vs. 10^3	-1544	-1645 to -1443	Yes	****	<0.0001
	0 vs. 10^2	-175.4	-289.4 to -61.47	Yes	*	0.015
E. coli O157 (Ec134)	0 vs. 10^7	-1012262	-1323465 to -701060	Yes	**	0.0049
	0 vs. 10^6	-367429	-378334 to -356524	Yes	****	<0.0001
	0 vs. 10^5	-29949	-31581 to -28317	Yes	***	0.0002
	0 vs. 10^4	-2811	-3499 to -2122	Yes	**	0.0031
	0 vs. 10^3	-144.8	-250.0 to -39.52	Yes	*	0.0207
	0 vs. 10^2	-79.78	-139.7 to -19.89	Yes	*	0.0186
E. coli O157 (Ec135)	0 vs. 10^7	-124998084	-138025201 to -11197096[illegible]	Yes	***	0.0006
	0 vs. 10^6	-7893750	-8206911 to -7580590	Yes	****	<0.0001
	0 vs. 10^5	-551617	-674566 to -428667	Yes	**	0.0026
	0 vs. 10^4	-42733	-48371 to -37096	Yes	***	0.0009
	0 vs. 10^3	-3959	-5227 to -2692	Yes	**	0.0053
	0 vs. 10^2	-424.4	-762.0 to -86.88	Yes	*	0.0321
E. coli O157 (Ec137)	0 vs. 10^7	-155298079	-170747325 to -13984883[illegible]	Yes	***	0.0005
	0 vs. 10^6	-8310078	-8571181 to -8048976	Yes	****	<0.0001
	0 vs. 10^5	-447012	-483027 to -410996	Yes	***	0.0003
	0 vs. 10^4	-26722	-29321 to -24123	Yes	***	0.0005
	0 vs. 10^3	-2498	-2752 to -2244	Yes	***	0.0005
	0 vs. 10^2	-194.8	-435.3 to 45.77	No	ns	0.0748

In the summary column, significance is indicated as *$P \leq 0.05$, **$P \leq 0.01$, ***$P \leq 0.001$, or ****$P \leq 0.0001$.

Supplementary Table 4: Statistical analysis of luminescence levels at different *E. coli* O157 pre-incubation times.

One-way ANOVA with Brown–Forsythe and Welch tests	Dunnett's T3 multiple comparisons test	Mean difference	95.00% CI of difference	Below threshold (0.05)?	Summary	Adjusted *P*-value
E. coli O157 (ATCC 43888)	Only phage vs. 0 h	-1444	-2033 to -854.7	Yes	**	0.0086
	Only phage vs. 1 h	53.67	-286.3 to 393.7	No	ns	0.8
	Only phage vs. 2 h	1115	-301.9 to 2531	No	ns	0.0791
	Only phage vs. 3 h	112	-393.3 to 617.3	No	ns	0.5972
	Only phage vs. 4 h	-15	-1514 to 1484	No	ns	>0.9999
	Only phage vs. 5 h	-13072	-22081 to -4062	Yes	*	0.0242
	Only phage vs. 6 h	-44060	-73372 to -14748	Yes	*	0.0226
	Only phage vs. 7 h	-221793	-383629 to -59957	Yes	*	0.027
	Only phage vs. 8 h	-1813884	-3520785 to -106983	Yes	*	0.0444

In the summary column, significance is indicated as *$P \leq 0.05$, **$P \leq 0.01$, ***$P \leq 0.001$, or ****$P \leq 0.0001$.

Supplementary Table 5: Phage defense systems searched in *E. coli* O157 clinical isolates.

E. coli O157 isolate			Defense systems (subtype)																		
Name	Clade	SG genotype	AbiD	AbiJ	AbiQ	CAS_Class1-Subtype-I-E	DarTG	Dnd_ABCDEFGH	Druantia_III	Lamassu-Fam	Mok_Hok_Sok	PD-T4-3	PsyrTA	Retron_VI	RM_Type_I	RM_Type_II	RM_Type_IIG	RnlAB	RosmerTA	Zorya_TypeI	Zorya_TypeII
E157-30	3	SG13				+	+		+	+	+	+	+		++	+	+	+		+	+
E157-13	3	SG13				+	+		+	+	+	+	+		++	+	+	+		+	+
E157-4	3	SG13				+	+		+	+	+	+	+		++	+	+	+		+	+
E157-48	3	SG13				+	+		+	+	++	+	+		++	+	+	+		+	+
E157-46	3	SG13				+	+		+	+	+	+	+		++	+	+	+		+	+
E157-5	3	SG13				+	+		+	+	+	+	+		++	+	+	+		+	+
E157-49	3	SG13				+	+		+	+	+	+	+		++	+	+	+		+	+
E157-47	3	SG13				+	+		+	+	+	+	+		++	+	+	+		+	+
E157-50	3	SG13				+	+		+	+	+	+	+		++	+	+			+	+
E157-28	3	SG13				+	+		+	+	+	+	+		++	+	+	+		+	+
Ec137	1	SG1				+			+	+	+	+	+		+	+	+	+			+
E157-20	1	SG4_6				+			+	+	+	+	+		+	+	+	+			+
Ec135	2	SG9				+			+	+	++	+	+		+	+	+				+
E157-25	2	SG9				+			+	+	+	+	+		+	+	+				+
E157-23	2	SG9				+			+	+	+	+	+		+	+	+		+		+
E157-17	2	SG9				+			+	+	+	+	+		+	+	+		+		+
E157-12	2	SG9				++			+	+	++	+	+		+	+	+				+
E157-7	2	SG9				+			+	+	++	+	+		+	+	+				+
E157-27	2	SG9				+			+	+	++	+	+		+	+	+				+
ATCC43888	4_5	SG16_17				+		+	+	+	+	+	+		+	+	+				+
Ec134	6	SG24				+			+	+	++	+	+		+	+	+		+		+
E157-22	4_5	SG20_23				+			+	+	+	+	+		+		+	+			+
E157-29	4_5	SG20_23				+	+		+	+	++	+	+		++	+	+			+	+
E157-45	7	SG28				+			+	+	++	+	+		+	+	+	+			+
E157-16	7	SG28				+			+	+	+	+	+		+	+	+	+			+
E157-21	7	SG28				+			+	+	+	+	+		+	+	+	+			+
E157-42	7	SG28				+			+	+	+	+	+		+	+	+	+			+
E157-39	7	SG28				+			+	+	+	+	+		+	+	+	+			+
E157-40	7	SG28				+			+	+	+	+	+		+	+	+	+			+
E157-6	7	SG28				+			+	+	+	+	+		+	+	+	+			+
E157-36	7	SG28				+			+	+	+	+	+		+	+	+	+			+
E157-1	7	SG28				+			+	+	+	+	+		+	+	+	+			+
E157-2	8	SG32				+			+	+	+	+	+		+	+	+				+
E157-14	8	SG31				+			+	++	+	+	+		+		+				+
E157-11	8	SG30				+			+	++	+	+	+		+		+				+
E157-26	8	SG30				+			+	++	++	+	+		++		+				+
E157-3	8	SG30				+			+	++	+	+	+		++		+				+
E157-18	7	SG29				+	+		+	++	+	+	+		++		+			+	+
E157-15	7	SG29				+	+		+	++	+	+	+		++		+			+	+
E157-41	7	SG29				+	+		+	++	+	+	+		++		+				+
E157-24	7	SG29				+	+		+	++	+	+	+		++		+			+	+
E157-10	7	SG29			+	+			+	+	+	+	+		+	+	+				+
E157-9	7	SG29			+	+			+	+	+		+		+	+	+		+		+
E157-44	7	SG29			+	+			+	+	+	+	+		+	+	+		+		+
E157-43	7	SG29				+			+	+	+	+	+		+	+	+				+
E157-37	7	SG29				+			+	+	+	+	+		+	+	+		+		+
E157-38	7	SG29				+			+	+	+		+	+	+	+	+				+
E157-31	9	UT				+			+	+	+		+		+		+				+
E157-34	9	UT				+			+	+	+		+		+		+				+
E157-35	9	UT				+			+	+	+		+		+		+				+
E157-33	9	UT				+			+	+	+		+		+		+				+
E157-32	9	UT	+			+			+	+	+		+		+		+				+
E157-8	9	UT		+		+			+	++	+		+		+		+				+

+; The *E. coli* O157 isolate possessed the corresponding defense system at a single site.

++; The *E. coli* O157 isolate possessed the corresponding defense system at two different sites.

Supplementary Table 6: Comparison of *E. coli* O157 detection methods.

Type of detection method	Preparation	Time required for detection	Detectability of viable cells	Detection accuracy	Accessibility	References
Culture-based detection	・Selective media<br>・Equipment and reagents for the subsequent biochemical or molecular analyses	Negative results take 3–7 days to obtain, and positive results take another 2 days or more to confirm.	It only detects viable cells.	Highly accurate and reliable	Inexpensive and simple	[4-7]
PCR-based detection	・Sample pretreatment, such as enrichment or genomic DNA extraction<br>・Thermal cyclers<br>・PCR primers, probes, DNA polymerase, or other reagents	Multiplex real-time PCR assays can detect 200 fg of *E. coli* O157 DNA (40 CFU/25 µL) within 2 h after overnight enrichment and DNA preparation.	It cannot distinguish between living and dead cells without the use of cell viability dye.	It is highly accurate, but DNA can only be amplified and detected from cells with intact membranes.	It requires expertise.	[6-9]
Phage particle adsorption-based detection	・Cloning of target genes (phage receptor binding proteins)<br>・Expression and purification of fluorescently fused proteins<br>・Spectrofluorometer	After bacterial pre-enrichment to 10^8 CFU/mL, phage fluorescent receptor binding proteins can detect *Enterococcus* and *Staphylococcus* in less than 1.5 h.	It relies on phage attachment and is unable to distinguish between living and dead cells.	It is specific but insensitive because it only uses phage proteins, with no phage propagation or reporter gene amplification.	Simple	[6, 10-11]
Fluorescent-labeled phage-based detection	・Labeled phages (30 min, unstable)<br>・Fluorescent microscope or flow cytometry	Labeled phages combined with immunomagnetic separation can detect 1–10^4 CFU/mL Shiga toxin-producing *E. coli,* including *E. coli* O157, in 8 h.	It only detects viable cells.	Low accuracy due to signal loss or light emission	It is inexpensive, but analysis requires expertise.	[6, 10, 12-14]
Reporter phage-based detection	・Reporter phages prepared by homologous recombination or recombineering (long insertion, inefficient, time-consuming, labor-intensive)<br>・Luminometer or other equipment and materials	*E. coli* O157 detection (including pre-enrichment)<br>・lambda::*luxI* 1 CFU/mL in 6–6.5 h<br>・phiV10::*luxAB* 10^6 CFU/mL in 1 h<br>・phiV10::*lux* 1 CFU/mL in 6 h<br>・phiV10::*nlux* 5 CFU in 7 h<br>・PP01::*luxI* 10^4 CFU/mL in 1 h, 10 CFU/mL in 4 h	It only detects viable cells.	It is highly specific and sensitive, but there is insufficient research on detectin accuracy against clinical isolates.	Simple	[6, 14-18]
HiBiT-tagged phage-based detection	・Tagged phages prepared by in vitro synthesis (short insertion of 33 bp, highly efficient, 2 days, stable at 4 °C)<br>・Luminometer<br>・Reagents for HiBiT detection	For detection of *E. coli* O157 at 10^2 CFU/mL or higher, a 2-hour co-incubation with phages is required, while lower concentrations necessitate a 5-hour bacterial pre-enrichment.	It only detects viable cells.	It is highly accurate, but sensitivity decreases against some strains due to phage low infectivity.	Simple	This study

Supplementary Table 7: Phage and bacterial strains used in this study.

	Strain	Clinical isolate name	Characteristic(s)	Detection assays	Source or reference
Phages	T1		Coliphage, Drexlerviridae (former Siphoviridae) family, 48.84 kb genome		NBRC 20001
	T2		Coliphage, Straboviridae (former Myoviridae) family, 163.83 kb genome		NBRC 20002
	T3		Coliphage, Autographiviridae (former Podoviridae) family, 38.21 kb genome		NBRC 20003
	T4		Coliphage, Straboviridae family, 168.90 kb genome		NBRC 20004
	T5		Coliphage, Demerecviridae (former Siphoviridae) family, 121.75 kb genome		NBRC 20005
	T6		Coliphage, Straboviridae family, 168.70 kb genome		NBRC 20006
	T7		Coliphage, Autographiviridae family, 39.94 kb genome		NBRC 20007
	SP15		Coliphage, Demerecviridae family, 110.96 kb genome		[19]
	PP01		*E. coli* O157 specific phage, Straboviridae family, 167.81 kb genome		[20]
	vB_Eco4M-7		*E. coli* O157 specific phage, Wifcevirus, 68.19 kb genome	Figs. 4, 5a	[21]
	O157_vB$_{HiBiT(gp19)}$		vB_Eco4M-7 with HiBiT tag at the C-terminus of gp19 (hypothetical protein)	Fig. 5a	This study
	O157_vB$_{HiBiT(gp20)}$		vB_Eco4M-7 with HiBiT tag at the C-terminus of gp20 (hypothetical protein)	Fig. 5a	This study
	O157_vB$_{HiBiT(gp27)}$		vB_Eco4M-7 with HiBiT tag at the C-terminus of gp27 (tail fiber protein)	Figs. 5a, 5b, 5c, 6a	This study
	O157_vB$_{HiBiT(gp32)}$		vB_Eco4M-7 with HiBiT tag at the C-terminus of gp32 (hypothetical protein)	Fig. 5a	This study
	O157_vB$_{HiBiT(gp35)}$		vB_Eco4M-7 with HiBiT tag at the C-terminus of gp35 (hypothetical protein)	Fig. 5a	This study
	O157_vB$_{HiBiT(gp36)}$		vB_Eco4M-7 with HiBiT tag at the C-terminus of gp36 (portal protein)	Fig. 5a	This study
	O157_vB$_{HiBiT(gp37)}$		vB_Eco4M-7 with HiBiT tag at the C-terminus of gp37 (minor capsid protein)	Figs. 4, 5a	This study
	O157_vB$_{HiBiT(gp55)}$		vB_Eco4M-7 with HiBiT tag at the C-terminus of gp55 (tail fiber protein)	Fig. 5a	This study
	O157_vB$_{HiBiT(gp64)}$		vB_Eco4M-7 with HiBiT tag at the C-terminus of gp64 (lytic enzyme)	Figs. 4, 5a	This study
	O157_vB$_{HiBiT(gp75)}$		vB_Eco4M-7 with HiBiT tag at the C-terminus of gp75 (DNA polymerase III alpha subunit)	Fig. 5a	This study
	O157_vB$_{HiBiT(gp85)}$		vB_Eco4M-7 with HiBiT tag at the C-terminus of gp85 (hypothetical protein)	Fig. 5a	This study
	O157_vB$_{HiBiT(gp86)}$		vB_Eco4M-7 with HiBiT tag at the C-terminus of gp86 (zinc independent RepB primase)	Fig. 5a	This study
	O157_vB$_{HiBiT(gp91)}$		vB_Eco4M-7 with HiBiT tag at the C-terminus of gp91 (DNA primase)	Fig. 5a	This study
Bacteria	*Escherichia coli* B		Laboratory strain	Fig. 4	NBRC 13168
	E. coli K-12 DH10B		Laboratory strain, Genotype: F^{-}, *mcrA*, Δ(*mrr-hsdRMS-mcrBC*), φ80*lacZ*ΔM15, Δ*lacX74*, *recA*1, *endA*1, *araD139*, Δ(*ara-leu*)7697, *galU*, *galK*, λ^{-}, *rpsL*(Str^{R}), *nupG*	Fig. 4	Thermo Fisher Scientific Inc. (Waltham, MA, USA)
	E. coli K-12 HST08		*E. coli* HST08 Premium Electro-Cells, Genotype: F^{-}, *endA*1, *supE*44, *thi*-1, *recA*1, *relA*1, *gyrA*96, *phoA*, φ80d*lacZ*ΔM15, Δ(*lacZYA-argF*)U169, Δ(*mrr-hsdRMS-mcrBC*), Δ*mcrA*, λ^{-}	Fig. 4	Takara Bio Inc. (Shiga, Japan)
	E. coli O157:H7		Shiga toxin-deficient human fecal isolate, Clade 4_5, SG genotype SG16_17	Figs. 4, 5a, 5b, 5c, 6a	ATCC 43888
	E. coli O157:H7		ATCC 43888 Δ*rfbE*::*cat*		NIID, [22]
	E. coli O157:H7		ATCC 43888 Δ*rfbE*::*cat*/pGEM-T-Easy-*rfbE*+		NIID, [22]
	E. coli O157:H7		Sakai, Shiga toxin (Stx) type 1, 2		NIID
	E. coli O157:H7		Sakai Δ*rfbE*::*kan*		NIID, [22]
	E. coli O121:H19	Ec133		Fig. 4	RIMD 05091859
	E. coli O157:H7	Ec134	Clade 6, SG genotype SG24	Figs. 4, 5a, 5b, 6a	RIMD 05091861
	E. coli O157:H7	Ec135	Clade 2, SG genotype SG9	Figs. 4, 5a, 5b, 6a	RIMD 05091909
	E. coli O111:HUT	Ec136		Fig. 4	RIMD 05092017
	E. coli O157:H7	Ec137	Sakai, Clade 1, SG genotype SG1	Figs. 4, 5a, 5b, 6a	RIMD 0509952
	E. coli ENec160852	Ec71	Cephem resistance, Quinolone resistance		Hibiki Research Group for Clinical Microbiology
	E. coli ENec160856	Ec72	Cephem resistance, Quinolone resistance		Hibiki Research Group for Clinical Microbiology
	E. coli Rec151	Ec81	Carbapenemase-Producing Enterobacteriaceae (CPE)		Hibiki Research Group for Clinical Microbiology
	E. coli Rec599	Ec82	CPE		Hibiki Research Group for Clinical Microbiology
	E. coli Rec6404	Ec89	CPE		Hibiki Research Group for Clinical Microbiology
	E. coli Mec4294	Ec104	CPE		Hibiki Research Group for Clinical Microbiology
	E. coli 3153A	Ec77	Extended-spectrum beta-lactamase (ESBL)-producing *E. coli*		Jichi Medical University
	E. coli 3563C	Ec78	ESBL-producing *E. coli*		Jichi Medical University
	E. coli 3594A	Ec138			Jichi Medical University
	E. coli O1	Ec147			Jichi Medical University
	E. coli O157	E157-1	Clade 7, SG genotype SG28	Fig. 6a	NIID
	E. coli O157	E157-2	Clade 8, SG genotype SG32	Fig. 6a	NIID
	E. coli O157	E157-3	Clade 8, SG genotype SG30	Fig. 6a	NIID
	E. coli O157	E157-4	Clade 3, SG genotype SG13	Fig. 6a	NIID
	E. coli O157	E157-5	Clade 3, SG genotype SG13	Fig. 6a	NIID
	E. coli O157	E157-6	Clade 7, SG genotype SG28	Fig. 6a	NIID
	E. coli O157	E157-7	Clade 2, SG genotype SG9	Fig. 6a	NIID
	E. coli O157	E157-8	Clade 9, SG genotype UT	Fig. 6a	NIID
	E. coli O157	E157-9	Clade 7, SG genotype SG29	Fig. 6a	NIID
	E. coli O157	E157-10	Clade 7, SG genotype SG29	Fig. 6a	NIID
	E. coli O157	E157-11	Clade 8, SG genotype SG30	Fig. 6a	NIID
	E. coli O157	E157-12	Clade 2, SG genotype SG9	Fig. 6a	NIID
	E. coli O157	E157-13	Clade 3, SG genotype SG13	Fig. 6a	NIID
	E. coli O157	E157-14	Clade 8, SG genotype SG31	Fig. 6a	NIID
	E. coli O157	E157-15	Clade 7, SG genotype SG29	Fig. 6a	NIID
	E. coli O157	E157-16	Clade 7, SG genotype SG28	Fig. 6a	NIID
	E. coli O157	E157-17	Clade 2, SG genotype SG9	Fig. 6a	NIID
	E. coli O157	E157-18	Clade 7, SG genotype SG29	Fig. 6a	NIID
	E. coli O157	E157-20	Clade 1, SG genotype SG4_6	Fig. 6a	NIID
	E. coli O157	E157-21	Clade 7, SG genotype SG28	Fig. 6a	NIID
	E. coli O157	E157-22	Clade 4_5, SG genotype SG20_23	Fig. 6a	NIID
	E. coli O157	E157-23	Clade 2, SG genotype SG9	Fig. 6a	NIID
	E. coli O157	E157-24	Clade 7, SG genotype SG29	Fig. 6a	NIID
	E. coli O157	E157-25	Clade 2, SG genotype SG9	Fig. 6a	NIID
	E. coli O157	E157-26	Clade 8, SG genotype SG30	Fig. 6a	NIID
	E. coli O157	E157-27	Clade 2, SG genotype SG9	Fig. 6a	NIID
	E. coli O157	E157-28	Clade 3, SG genotype SG13	Fig. 6a	NIID
	E. coli O157	E157-29	Clade 4_5, SG genotype SG20_23	Fig. 6a	NIID
	E. coli O157	E157-30	Clade 3, SG genotype SG13	Fig. 6a	NIID
	E. coli O157	E157-31	Clade 9, SG genotype UT	Fig. 6a	NIID
	E. coli O157	E157-32	Clade 9, SG genotype UT	Fig. 6a	NIID
	E. coli O157	E157-33	Clade 9, SG genotype UT	Fig. 6a	NIID
	E. coli O157	E157-34	Clade 9, SG genotype UT	Fig. 6a	NIID
	E. coli O157	E157-35	Clade 9, SG genotype UT	Fig. 6a	NIID
	E. coli O157	E157-36	Clade 7, SG genotype SG28	Fig. 6a	NIID
	E. coli O157	E157-37	Clade 7, SG genotype SG29	Fig. 6a	NIID
	E. coli O157	E157-38	Clade 7, SG genotype SG29	Fig. 6a	NIID
	E. coli O157	E157-39	Clade 7, SG genotype SG28	Fig. 6a	NIID

E. coli O157	E157-40	Clade 7, SG genotype SG28	Fig. 6a	NIID
E. coli O157	E157-41	Clade 7, SG genotype SG29	Fig. 6a	NIID
E. coli O157	E157-42	Clade 7, SG genotype SG28	Fig. 6a	NIID
E. coli O157	E157-43	Clade 7, SG genotype SG29	Fig. 6a	NIID
E. coli O157	E157-44	Clade 7, SG genotype SG29	Fig. 6a	NIID
E. coli O157	E157-45	Clade 7, SG genotype SG28	Fig. 6a	NIID
E. coli O157	E157-46	Clade 3, SG genotype SG13	Fig. 6a	NIID
E. coli O157	E157-47	Clade 3, SG genotype SG13	Fig. 6a	NIID
E. coli O157	E157-48	Clade 3, SG genotype SG13	Fig. 6a	NIID
E. coli O157	E157-49	Clade 3, SG genotype SG13	Fig. 6a	NIID
E. coli O157	E157-50	Clade 3, SG genotype SG13	Fig. 6a	NIID
E. coli O26:H11	E26-1	Stx type 2	Fig. 6a	NIID
E. coli O26:H11	E26-2	Stx type 1	Fig. 6a	NIID
E. coli O26:H-	E26-3	Stx type 1, 2	Fig. 6a	NIID
E. coli O103:H11	E103-1	Stx type 1	Fig. 6a	NIID
E. coli O103:H2	E103-2	Stx type 1	Fig. 6a	NIID
E. coli O103:H-/Hg8	E103-3	Stx type 1	Fig. 6a	NIID
E. coli O103:H25	E103-4	Stx type 1	Fig. 6a	NIID
E. coli O111:H-/Hg8	E111-1	Stx type 1, 2	Fig. 6a	NIID
E. coli O111:H21	E111-2		Fig. 6a	NIID
E. coli O111:H-/Hg8	E111-3	Stx type 1	Fig. 6a	NIID
E. coli O111:H-/Hg8	E111-4	Stx type 2	Fig. 6a	NIID
E. coli O121:H19	E121-1	Stx type 2	Fig. 6a	NIID
E. coli O121:H19	E121-2	Stx type 2	Fig. 6a	NIID
E. coli O121:H10	E121-3	Stx type 2	Fig. 6a	NIID
E. coli O145:H-/Hg28	E145-1	Stx type 2	Fig. 6a	NIID
E. coli O145:H-/Hg28	E145-2	Stx type 1	Fig. 6a	NIID
E. coli O145:H34	E145-3	Stx type 2	Fig. 6a	NIID
E. coli O91:H14	E91-1	Stx type 1	Fig. 6a	NIID
E. coli O91:H14	E91-2	Stx type 1, 2	Fig. 6a	NIID
E. coli O91:H-/Hg14	E91-3	Stx type 1	Fig. 6a	NIID
E. coli O165:H-/Hg25	E165-1	Stx type 1, 2	Fig. 6a	NIID
E. coli O165:H-/Hg25	E165-2	Stx type 2	Fig. 6a	NIID
E. coli O165:H-/Hg25	E165-3	Stx type 1, 2	Fig. 6a	NIID
E. coli O115:H1/H12	E115-1	Stx type 2	Fig. 6a	NIID
E. coli O115:H10	E115-2	Stx type 1	Fig. 6a	NIID
E. coli O115:H-/Hg25	E115-3	Stx type 2	Fig. 6a	NIID
E. coli O156:H-/Hg25	E156-1	Stx type 1	Fig. 6a	NIID
E. coli O156:H25	E156-2	Stx type 1	Fig. 6a	NIID
E. coli O156:H-/Hg25	E156-3	Stx type 1	Fig. 6a	NIID
E. coli O5:H-/Hg9	E5-1	Stx type 1	Fig. 6a	NIID
E. coli O5:H-/Hg19	E5-2	Stx type 1, 2	Fig. 6a	NIID
E. coli O5:H-/Hg9	E5-3	Stx type 1	Fig. 6a	NIID
E. coli O172:H-/Hg25	E172-1	Stx type 2	Fig. 6a	NIID
E. coli O172:H-/Hg25	E172-2	Stx type 2	Fig. 6a	NIID
E. coli O172:H-/Hg25	E172-3	Stx type 2	Fig. 6a	NIID

NBRC: National Institute of Technology and Evaluation (NITE) Biological Resource Center

ATCC: American Type Culture Collection

RIMD: Research Institute for Microbial Diseases, Osaka University

NIID: National Institute of Infectious Diseases, Japan

Supplementary Table 8: PCR primers used in this study.

Primer used	Primer name	Sequence (5' to 3')
Synthesis of T7 phages	T7-20-1f	CCGTCAACAAGTGCTGGAAGATACCATTCAGCCAG
	T7-20-1r	AGAATACGAACGAGGACAAGGTTAGGCATATCGCC
	T7-20-2f	CTTGTCCTCGTTCGTATTCTCAAGTGCCGCTTTAC
	T7-20-2r	TCGGGATGTATGGGCAAGCCTCTTTAGGATAAGTC
	T7-20-3f	GGCTTGCCCATACATCCCGATTCGGATGGTCAGAC
	T7-20-3r	CAGATGGGATTGCGCCAGTAACCAGACCGTTGTCG
	T7-20-4f	TACTGGCGCAATCCCATCTGATGCTCAAGCCACAC
	T7-20-4r	CGTCCATTGAGTCTGACCCTAGATGCCATCCTAAC
	T7-20-5f	AGGGTCAGACTCAATGGACGCTAAATGGAAACAGG
	T7-20-5r	CTTCCAGCACTTGTTGACGGAAGCCGAACTCTTTG
	T7-40-1f	GCTTCCGTCAACAAGTGCTGGAAGATACCATTCAG
	T7-40-1r	CGAACGAGGACAAGGTTAGGCATATCGCCTTGCTG
	T7-40-2f	GTAATCAGCAAGGCGATATGCCTAACCTTGTCCTC
	T7-40-2r	TGTACGAACGACCGTAGGATTCACCATCTAGTCTG
	T7-40-3f	ATGGTCAGACTAGATGGTGAATCCTACGGTCGTTC
	T7-40-3r	TTGAGCATCAGATGGGATTGCGCCAGTAACCAGAC
	T7-40-4f	CAACGGTCTGGTTACTGGCGCAATCCCATCTGATG
	T7-40-4r	TTAGCGTCCATTGAGTCTGACCCTAGATGCCATCC
	T7-40-5f	GGTTAGGATGGCATCTAGGGTCAGACTCAATGGAC
	T7-40-5r	GCTGGCTGAATGGTATCTTCCAGCACTTGTTGACG
	T7-60-1f	AGTTCGGCTTCCGTCAACAAGTGCTGGAAGATACC
	T7-60-1r	CGGCACTTGAGAATACGAACGAGGACAAGGTTAGG
	T7-60-2f	TGAGCGTAATCAGCAAGGCGATATGCCTAACCTTG
	T7-60-2r	GGGATGTATGGGCAAGCCTCTTTAGGATAAGTCCC
	T7-60-3f	GGGTATGGAAGTCCAAGGCTCCGATGGGACTTATC
	T7-60-3r	TTGGCTTATAAGCTGTGTGGCTTGAGCATCAGATG
	T7-60-4f	AACGGTCTGGTTACTGGCGCAATCCCATCTGATGC
	T7-60-4r	CGTCCATTGAGTCTGACCCTAGATGCCATCCTAAC
	T7-60-5f	GGGTTCTCGCTGGGATGCTATTCGGGTTAGGATGG
	T7-60-5r	TGCCGGAATCAATAGCTGGCTGAATGGTATCTTCC
	T7-80-1f	GTTCAGTCATGACGCTGGCTTACGGGTCCAAAGAG
	T7-80-1r	CACCAGTAAAGCGGCACTTGAGAATACGAACGAGG
	T7-80-2f	TATTGCCCTTGAGCGTAATCAGCAAGGCGATATGC
	T7-80-2r	AGTCACCTAAGTATTCCTCAATGTACGAACGACCG
	T7-80-3f	CCCATACATCCCGATTCGGATGGTCAGACTAGATG
	T7-80-3r	ACTGAACGCTTGGCTTATAAGCTGTGTGGCTTGAG
	T7-80-4f	AGTATATCGACAACGGTCTGGTTACTGGCGCAATC
	T7-80-4r	TCCTGTTTCCATTTAGCGTCCATTGAGTCTGACCC
	T7-80-5f	CCTTGGGTTCTCGCTGGGATGCTATTCGGGTTAGG
	T7-80-5r	AGCTGGCTGAATGGTATCTTCCAGCACTTGTTGAC
	T7-100-1f	CTCGCAGTGTGACTAAGCGTTCAGTCATGACGCTG
	T7-100-1r	CATCCGGTTTCCTTGTTGTATTCCATGTAGCCAGC
	T7-100-2f	GCGATATGCCTAACCTTGTCCTCGTTCGTATTCTC
	T7-100-2r	AACGACCGTAGGATTCACCATCTAGTCTGACCATC
	T7-100-3f	GGTATGGAAGTCCAAGGCTCCGATGGGACTTATCC
	T7-100-3r	GTTACCTTCTTGTCACCGACTCGCATCAGGAAGTC
	T7-100-4f	ATGCTCAAGCCACACAGCTTATAAGCCAAGCGTTC
	T7-100-4r	TTTGAGTGCTCTTCGCAGCCTCAACTCTCTTAACG
	T7-100-5f	TTAGGATGGCATCTAGGGTCAGACTCAATGGACGC
	T7-100-5r	ATAGCTGGCTGAATGGTATCTTCCAGCACTTGTTG
Synthesis of O157_vB phages	O157_vB-1f	GTCAATGGCCGAGTGATTCACGTGTCGCACGACGC
	O157_vB-1r	GCGTACTATTTTCGGGCGAAGGGTCGGCATATTTC
	O157_vB-2f	AACGAGAAATATGCCGACCCTTCGCCCGAAAATAG
	O157_vB-2r	CTTCTGCTGTATCTTCTGGCAAACGGTTGTAGCCG
	O157_vB-3f	AACAGCGGCTACAACCGTTTGCCAGAAGATACAGC
	O157_vB-3r	AGTGCGGTATAGTATGAGCCAATGTTGGCGAATGC
	O157_vB-4f	CGACTGCATTCGCCAACATTGGCTCATACTATACC
	O157_vB-4r	GCGCTGCCTGTCCGCTTTTTACTGCTGCAATTTTG
	O157_vB-5f	GATTGCAAAATTGCAGCAGTAAAAAGCGGACAGGC
	O157_vB-5r	GCAGCGCATCAGTGAAATCAAAACTGTTCTGCTTC
	O157_vB-6f	GCCAGGAAGCAGAACAGTTTTGATTTCACTGATGC
	O157_vB-6r	AGATTGTCGACATTCCGAAAGACTTACGACACGCC
	O157_vB-7f	AGTAGGGCGTGTCGTAAGTCTTTCGGAATGTCGAC
	O157_vB-7r	GTGCGACACGTGAATCACTCGGCCATTGACAGGAATTG
Synthesis of O157_vB phages with a HiBiT tag	HiBiT-gp37-f	GTCTCCGGATGGCGCCTTTTTAAGAAAATCTCGTGAGGTCATTATGACGATACCAATCAACACATTCCC
	HiBiT-gp37-r	CGAGATTTTCTTAAAAAGGCGCCATCCGGAGACGATAATTGGTATTTTTCTGCACTTGCAGTGTATCGCC
	HiBiT-gp64-f	GTCTCCGGATGGCGCCTTTTTAAGAAAATCTCGTAAGAAAAAGGCCCATATGGGCCTTTGTTTATTTTAC
	HiBiT-gp64-r	CGAGATTTTCTTAAAAAGGCGCCATCCGGAGACCACAACTGCTAGTGCATCCTTGTAACGCTTCC
Synthesis of O157_vB phages with a HiBiT tag at various proteins	O157_vB-1f-2	GCCATCGGTAATACGACGGGGAAGGTTGCAAC
	O157_vB-1r-2	GACCGTAAGTGTATGCAAAAGAAGATTCGGCAAGC
	O157_vB-2f-2	GTGCTGAAGGTCGAGTTTTCTTGCAGCAAATG
	O157_vB-2r-2	CGACATCTGTCATATACACCTCAAGTAGACACTGG
	O157_vB-3f-2	AAACGACATGGTTTGACCAAGATGGTTGGACG
	O157_vB-3r-2	CTTGTGCAGGAATCATGCCGTCAGTGGTATACTTG
	O157_vB-4f-2	CCCAACCAAACTGACAAATCAAGTATGTTTGCCGC
	O157_vB-4r-2	TTAACATCGACCGCAGCAAGCAGGAAGAAG
	O157_vB-5f-2	GTCGATTAAAACTCTTCTGCGTCGGTCGGAGTTTC
	O157_vB-5r-2	CATGCTTGACACCATCAAGTCACCTTTCGACTTTG

	O157_vB-6f-2	GTGTGCGCACCATCTGATGAGTCATATTCTTTG
	O157_vB-6r-2	GGCCTGCATATTGCGTACATACTTCTGCTCTAATG
	O157_vB-7f-2	GAATACAGGCTTCACCATGTATGAATGGCACAC
	O157_vB-7r-2	GGTCGTAAATTGTTACGTAAGGTGACTAAAAGATGAC
	HiBiT-gp19-f	CGAGATTTTCTTAAAAAGGCGCCATCCGGAGACGATAATCTGGAAGGTGTAGGACTTGTTGCCGAACTCG
	HiBiT-gp19-r	GTCTCCGGATGGCGCCTTTTTAAGAAAATCTCGTAATCGAATGGCGGCCCTTCGGGGTCGCTTTTCATAG
	HiBiT-gp20-f	GTCTCCGGATGGCGCCTTTTTAAGAAAATCTCGTAACGAAAAAGTGCTTTACTTCTTCGTACACTAAGAC
	HiBiT-gp20-r	CGAGATTTTCTTAAAAAGGCGCCATCCGGAGACTTTTGCGATAAAATATTTATAAGCCTTATCGCCTTC
	HiBiT-gp27-f	CGAGATTTTCTTAAAAAGGCGCCATCCGGAGACTTTTGCTGCTTTCAGCGCGGCTATCTCTGCCTC
	HiBiT-gp27-r	GTCTCCGGATGGCGCCTTTTTAAGAAAATCTCGTAATTCACTCAAGGCGCTGAAAAGCGCCTTTAC
	HiBiT-gp32-f	CGAGATTTTCTTAAAAAGGCGCCATCCGGAGACGTGTGAAGACACAATTAGTGTGCCACTTCCCTTTG
	HiBiT-gp32-r	GTCTCCGGATGGCGCCTTTTTAAGAAAATCTCGTGATACAATGCCATGGACGGCATAATTCACAAAAAG
	HiBiT-gp33-f	GTCTCCGGATGGCGCCTTTTTAAGAAAATCTCGTAACAATCAAAGCATTCCGTGCTGAAGGTCGAG
	HiBiT-gp33-r	CGAGATTTTCTTAAAAAGGCGCCATCCGGAGACAAGGTTAGAGAAGAAGCCGGATGCTGCAACG
	HiBiT-gp35-f	CGAGATTTTCTTAAAAAGGCGCCATCCGGAGACTTTGATAATCCCCTTGATGTCTGATTCTGCCATAAAG
	HiBiT-gp35-r	GTCTCCGGATGGCGCCTTTTTAAGAAAATCTCGTGAGAACGTTAACCGAACTCGATGTTTTGAAAGATG
	HiBiT-gp36-f	GTCTCCGGATGGCGCCTTTTTAAGAAAATCTCGTGATATGGCTTTTAAAGCTTCGAAAAAACGTGAACGC
	HiBiT-gp36-r	CGAGATTTTCTTAAAAAGGCGCCATCCGGAGACACCTTGCTTCGGTGAATTAGTAGGATTATTGAGTGC
	HiBiT-gp37-f-2	GTCTCCGGATGGCGCCTTTTTAAGAAAATCTCGTGAGGTCATTATGACGATACCAATCAACACATTCCC
	HiBiT-gp37-r-2	CGAGATTTTCTTAAAAAGGCGCCATCCGGAGACGATAATTGGTATTTTTCTGCACTTGCAGTGTATCGCC
	HiBiT-gp41-f	GTCTCCGGATGGCGCCTTTTTAAGAAAATCTCGTAATAGGCAGGTGAATAATGACTCGCATTAGCCAAAC
	HiBiT-gp41-r	CGAGATTTTCTTAAAAAGGCGCCATCCGGAGACACGAGTCAATTGTACAATGGTATAAGTGGAAGACTGG
	HiBiT-gp42-f	GTCTCCGGATGGCGCCTTTTTAAGAAAATCTCGTAATTGCTGTGATTACACAGTAAAACACATGGGGCG
	HiBiT-gp42-r	CGAGATTTTCTTAAAAAGGCGCCATCCGGAGACAATACCAGTCATACGGACGACTGCGAAAGGAC
	HiBiT-gp48-f	GTCTCCGGATGGCGCCTTTTTAAGAAAATCTCGTAAATAATTGCCGCCGTAACTGGCGGCCATC
	HiBiT-gp48-r	CGAGATTTTCTTAAAAAGGCGCCATCCGGAGACAATAAGAACGTCACGACCTTCAACGCGACG
	HiBiT-gp55-f	GTCTCCGGATGGCGCCTTTTTAAGAAAATCTCGTAAAATGAAGATCACCAACAAACAAACTGGTCGTAC
	HiBiT-gp55-r	CGAGATTTTCTTAAAAAGGCGCCATCCGGAGACTATTCCTACTGTTGATTTTATTTTATTATATAACG
	HiBiT-gp57-f	GTCTCCGGATGGCGCCTTTTTAAGAAAATCTCGTGATAATTTGAAAAAGAGAATCCTTAAAGTCACC
	HiBiT-gp57-r	CGAGATTTTCTTAAAAAGGCGCCATCCGGAGACCCACTTCAAAGGTGTAGCAGTAAGGTTGTTTATGTCG
	HiBiT-gp62-f	GTCTCCGGATGGCGCCTTTTTAAGAAAATCTCGTAGAGGAACGGCAATGCCAACACTTATTAACAGACC
	HiBiT-gp62-r	CGAGATTTTCTTAAAAAGGCGCCATCCGGAGACCACGATGTATTTAATTTTTGATAAAAGGGTTGATGC
	HiBiT-gp64-f-2	GTCTCCGGATGGCGCCTTTTTAAGAAAATCTCGTAAGAAAAAGGCCCATATGGGCCTTTGTTTATTTTAC
	HiBiT-gp64-r-2	CGAGATTTTCTTAAAAAGGCGCCATCCGGAGACCACAACTGCTAGTGCATCCTTGTAACGCTTCC
	HiBiT-gp69-f	CGAGATTTTCTTAAAAAGGCGCCATCCGGAGACTTTTTCCTTTGATGCATTGAATTGATCG
	HiBiT-gp69-r	GTCTCCGGATGGCGCCTTTTTAAGAAAATCTCGTAAATGAAACAGATTTATGCAAACATTCGCCAGTGGG
	HiBiT-gp75-f	CGAGATTTTCTTAAAAAGGCGCCATCCGGAGACTTTGTTGCCATCTGGATACACCTGTTTGATAACGTCG
	HiBiT-gp75-r	GTCTCCGGATGGCGCCTTTTTAAGAAAATCTCGTAACCGTGAACAAAAATACATTTTCGTGCCCCTGAAG
	HiBiT-gp85-f	CGAGATTTTCTTAAAAAGGCGCCATCCGGAGACTAACTCCACTACGCTGTCAAGGTCCAGATATACTGG
	HiBiT-gp85-r	GTCTCCGGATGGCGCCTTTTTAAGAAAATCTCGTAATGAACATTCCAGAATTTAATGATGAGCCAG
	HiBiT-gp86-f	GTCTCCGGATGGCGCCTTTTTAAGAAAATCTCGTAGTTTGCTTTTATTTTCATAGCAACTATAATC
	HiBiT-gp86-r	CGAGATTTTCTTAAAAAGGCGCCATCCGGAGACTTTTATGCCACCTGCACGACGATTTATCATGTCAAG
	HiBiT-gp91-f	GTCTCCGGATGGCGCCTTTTTAAGAAAATCTCGTGATGAATAAAGCAATCAACATCGCTAAATTCTTTC
	HiBiT-gp91-r	CGAGATTTTCTTAAAAAGGCGCCATCCGGAGACGAATTCATCCTTATCTCTTCCCAAAGAACGTTTACGC
HiBiT amplification and sequencing	HiBiT Check-gp19-f	CGCCAGCAAGTCTCGAATAG
	HiBiT Check-gp19-r	CGCCGTTCGAATAGCAAAGG
	HiBiT Check-gp20-f	CCGTTAACGCGGAACTAGAATC
	HiBiT Check-gp20-r	GGCTCGATAGTTGCGTCTAC
	HiBiT Check-gp27-f	CATCGCCTGTAACGGCTTTC
	HiBiT Check-gp27-r	CCGTCGGTTTTGGCTTCATC
	HiBiT Check-gp32-f	ACCAGATAGACCAGCACAAGATG
	HiBiT Check-gp32-r	CCATGGCCCTAGTCAACATTC
	HiBiT Check-gp35-f	AAATGACCTGGCACGGAATC
	HiBiT Check-gp35-r	TTTTAGGTCGCAAGGTGTATCC
	HiBiT Check-gp36-f	CTGGCGAATTTGATGAACACAAG
	HiBiT Check-gp36-r	GAGTCTTGCGCAAAGAACTTTTTC
	HiBiT Check-gp37-f	TGCGACAGAATGGCGTTGAG
	HiBiT Check-gp37-r	CTGGTCGAATCGCCATCATTG
	HiBiT Check-gp48-f	GGCACCATCTCTGTCGGTAAG
	HiBiT Check-gp48-r	GGTTTGCACGGTTCCATACTAC
	HiBiT Check-gp55-f	TCGATGTCCGACGTTGAAATAAC
	HiBiT Check-gp55-r	CCAGTCACCAAGATGGTTGTC
	HiBiT Check-gp62-f	CAACAAGCGAAGATTTCGCAAAG
	HiBiT Check-gp62-r	AGGTTGTCGGTGATCAGATTCAG
	HiBiT Check-gp64-f	TGACTGGAAAGGCCAACTATGC
	HiBiT Check-gp64-r	GCTTTGACGTCTCAAATCTTGTC
	HiBiT Check-gp75-f	GCTGCATTGACCTTTCTCAAATG
	HiBiT Check-gp75-r	TTGACCAGTACGGGAACAAG
	HiBiT Check-gp85-f	GCAATATAGATAGCGCCGTACTC
	HiBiT Check-gp85-r	ATGCGATTCCTGGGTCAAC
	HiBiT Check-gp86-f	ATCTGTCCTTGGGGTGAAGAG
	HiBiT Check-gp86-r	CTTCAACGGTCATCATTTTTGG
	HiBiT Check-gp91-f	ACATGTCGAAGCTTGGTTTTGAG
	HiBiT Check-gp91-r	GGGTCAATGATCATCACATCTTTCC

Supplementary Table 9: PCR conditions for amplification of phage DNA fragments.

Phage	Fragment No.	Primer 1	Primer 2	Template	Fragment length (bp)	DNA polymerase	Reaction volume (µL)	Denature temperature (°C)	Denature time (s)	Annealing temperature (°C)	Annealing time (s)	Extension temperature (°C)	Extension time (min)	Cycles
T7	1	T7-40-1f	T7-40-1r	T7 genome	8,011	KOD FX Neo (Toyobo Co., Ltd., Osaka, Japan)	50	98	10	-	-	68	10	40
	2	T7-40-2f	T7-40-2r	T7 genome	7,970	KOD FX Neo	50	98	10	-	-	68	10	40
	3	T7-40-3f	T7-40-3r	T7 genome	8,037	KOD FX Neo	50	98	10	-	-	68	10	40
	4	T7-40-4f	T7-40-4r	T7 genome	8,010	KOD FX Neo	50	98	10	-	-	68	10	40
	5	T7-40-5f	T7-40-5r	T7 genome	7,949	KOD FX Neo	50	98	10	-	-	68	10	40
T7 (fragment overlaps: 20 bp)	1	T7-20-1f	T7-20-1r	T7 genome	8,013	KOD FX Neo	50	98	10	-	-	68	10	40
	2	T7-20-2f	T7-20-2r	T7 genome	7,899	KOD FX Neo	50	98	10	-	-	68	10	40
	3	T7-20-3f	T7-20-3r	T7 genome	8,054	KOD FX Neo	50	98	10	-	-	68	10	40
	4	T7-20-4f	T7-20-4r	T7 genome	7,994	KOD FX Neo	50	98	10	-	-	68	10	40
	5	T7-20-5f	T7-20-5r	T7 genome	7,917	KOD FX Neo	50	98	10	-	-	68	10	40
T7 (fragment overlaps: 60 bp)	1	T7-60-1f	T7-60-1r	T7 genome	8,032	KOD FX Neo	50	98	10	-	-	68	10	40
	2	T7-60-2f	T7-60-2r	T7 genome	7,928	KOD FX Neo	50	98	10	-	-	68	10	40
	3	T7-60-3f	T7-60-3r	T7 genome	8,125	KOD FX Neo	50	98	10	-	-	68	10	40
	4	T7-60-4f	T7-60-4r	T7 genome	8,005	KOD FX Neo	50	98	10	-	-	68	10	40
	5	T7-60-5f	T7-60-5r	T7 genome	7,987	KOD FX Neo	50	98	10	-	-	68	10	40
T7 (fragment overlaps: 80 bp)	1	T7-80-1f	T7-80-1r	T7 genome	8,076	KOD FX Neo	50	98	10	-	-	68	10	40
	2	T7-80-2f	T7-80-2r	T7 genome	8,005	KOD FX Neo	50	98	10	-	-	68	10	40
	3	T7-80-3f	T7-80-3r	T7 genome	8,086	KOD FX Neo	50	98	10	-	-	68	10	40
	4	T7-80-4f	T7-80-4r	T7 genome	8,032	KOD FX Neo	50	98	10	-	-	68	10	40
	5	T7-80-5f	T7-80-5r	T7 genome	7,978	KOD FX Neo	50	98	10	-	-	68	10	40
T7 (fragment overlaps: 100 bp)	1	T7-100-1f	T7-100-1r	T7 genome	8,140	KOD FX Neo	50	98	10	-	-	68	10	40
	2	T7-100-2f	T7-100-2r	T7 genome	7,952	KOD FX Neo	50	98	10	-	-	68	10	40
	3	T7-100-3f	T7-100-3r	T7 genome	8,195	KOD FX Neo	50	98	10	-	-	68	10	40
	4	T7-100-4f	T7-100-4r	T7 genome	8,040	KOD FX Neo	50	98	10	-	-	68	10	40
	5	T7-100-5f	T7-100-5r	T7 genome	7,950	KOD FX Neo	50	98	10	-	-	68	10	40
O157_vB	1	O157_vB-1f	O157_vB-1r	O157_vB genome	9,629	KOD FX Neo	50	98	10	-	-	68	10	40
	2	O157_vB-2f	O157_vB-2r	O157_vB genome	9,824	KOD FX Neo	50	98	10	-	-	68	10	40
	3	O157_vB-3f	O157_vB-3r	O157_vB genome	9,806	KOD FX Neo	50	98	10	-	-	68	10	40
	4	O157_vB-4f	O157_vB-4r	O157_vB genome	9,743	KOD FX Neo	50	98	10	-	-	68	10	40
	5	O157_vB-5f	O157_vB-5r	O157_vB genome	9,802	KOD FX Neo	50	98	10	-	-	68	10	40
	6	O157_vB-6f	O157_vB-6r	O157_vB genome	9,734	KOD FX Neo	50	98	10	-	-	68	10	40
	7	O157_vB-7f	O157_vB-7r	O157_vB genome	9,816	KOD FX Neo	50	98	10	-	-	68	10	40
O157_vB$_{\mathrm{HiBiT(gp37)}}$	1	O157_vB-1f	O157_vB-1r	O157_vB genome	9,629	KOD FX Neo	50	98	10	-	-	68	10	40
	2	O157_vB-2f	O157_vB-2r	O157_vB genome	9,824	KOD FX Neo	50	98	10	-	-	68	10	40
	gp37-1	O157_vB-3f	HiBiT-gp37-r	O157_vB genome	1,783	KOD FX Neo	50	98	10	-	-	68	2	40
	gp37-2	HiBiT-gp37-f	O157_vB-3r	O157_vB genome	8,089	KOD FX Neo	50	98	10	-	-	68	10	40
	4	O157_vB-4f	O157_vB-4r	O157_vB genome	9,743	KOD FX Neo	50	98	10	-	-	68	10	40
	5	O157_vB-5f	O157_vB-5r	O157_vB genome	9,802	KOD FX Neo	50	98	10	-	-	68	10	40
	6	O157_vB-6f	O157_vB-6r	O157_vB genome	9,734	KOD FX Neo	50	98	10	-	-	68	10	40
	7	O157_vB-7f	O157_vB-7r	O157_vB genome	9,816	KOD FX Neo	50	98	10	-	-	68	10	40
O157_vB$_{\mathrm{HiBiT(gp64)}}$	1	O157_vB-1f	O157_vB-1r	O157_vB genome	9,629	KOD FX Neo	50	98	10	-	-	68	10	40
	2	O157_vB-2f	O157_vB-2r	O157_vB genome	9,824	KOD FX Neo	50	98	10	-	-	68	10	40
	3	O157_vB-3f	O157_vB-3r	O157_vB genome	9,806	KOD FX Neo	50	98	10	-	-	68	10	40
	4	O157_vB-4f	O157_vB-4r	O157_vB genome	9,743	KOD FX Neo	50	98	10	-	-	68	10	40
	gp64-1	O157_vB-5f	HiBiT-gp64-r	O157_vB genome	5,361	KOD FX Neo	50	98	10	-	-	68	5	40
	gp64-2	HiBiT-gp64-f	O157_vB-5r	O157_vB genome	4,507	KOD FX Neo	50	98	10	-	-	68	5	40
	6	O157_vB-6f	O157_vB-6r	O157_vB genome	9,734	KOD FX Neo	50	98	10	-	-	68	10	40
	7	O157_vB-7f	O157_vB-7r	O157_vB genome	9,816	KOD FX Neo	50	98	10	-	-	68	10	40
O157_vB, Second	1	O157_vB-1f-2	O157_vB-1r-2	O157_vB genome	10,033	KOD FX Neo	50	98	10	64	30	68	11	40
	2	O157_vB-2f-2	O157_vB-2r-2	O157_vB genome	10,168	KOD FX Neo	50	98	10	64	30	68	11	40
	3	O157_vB-3f-2	O157_vB-3r-2	O157_vB genome	9,944	KOD FX Neo	50	98	10	64	30	68	11	40
	4	O157_vB-4f-2	O157_vB-4r-2	O157_vB genome	10,379	KOD FX Neo	50	98	10	64	30	68	11	40
	5	O157_vB-5f-2	O157_vB-5r-2	O157_vB genome	10,187	KOD FX Neo	50	98	10	64	30	68	11	40
	6	O157_vB-6f-2	O157_vB-6r-2	O157_vB genome	9,252	KOD FX Neo	50	98	10	64	30	68	11	40
	7	O157_vB-7f-2	O157_vB-7r-2	O157_vB genome	8,802	KOD FX Neo	50	98	10	64	30	68	11	40
O157_vB$_{\mathrm{HiBiT(gp19)}}$	gp19-1	O157_vB-1f-2	HiBiT-gp19-r	O157_vB genome	381	KOD FX Neo	50	98	10	-	-	68	1	40
	gp19-2	HiBiT-gp19-f	O157_vB-1r-2	O157_vB genome	9,652	KOD FX Neo	50	98	10	64	30	68	11	40
	2	O157_vB-2f-2	O157_vB-2r-2	O157_vB genome	10,168	KOD FX Neo	50	98	10	64	30	68	11	40
	3	O157_vB-3f-2	O157_vB-3r-2	O157_vB genome	9,944	KOD FX Neo	50	98	10	64	30	68	11	40
	4	O157_vB-4f-2	O157_vB-4r-2	O157_vB genome	10,379	KOD FX Neo	50	98	10	64	30	68	11	40
	5	O157_vB-5f-2	O157_vB-5r-2	O157_vB genome	10,187	KOD FX Neo	50	98	10	64	30	68	11	40
	6	O157_vB-6f-2	O157_vB-6r-2	O157_vB genome	9,252	KOD FX Neo	50	98	10	64	30	68	11	40
	7	O157_vB-7f-2	O157_vB-7r-2	O157_vB genome	8,802	KOD FX Neo	50	98	10	64	30	68	11	40
O157_vB$_{\mathrm{HiBiT(gp20)}}$	gp20-1	O157_vB-1f-2	HiBiT-gp20-r	O157_vB genome	1,530	KOD FX Neo	50	98	10	65	30	68	2	40
	gp20-2	HiBiT-gp20-f	O157_vB-1r-2	O157_vB genome	8,503	KOD FX Neo	50	98	10	64	30	68	11	40
	2	O157_vB-2f-2	O157_vB-2r-2	O157_vB genome	10,168	KOD FX Neo	50	98	10	64	30	68	11	40
	3	O157_vB-3f-2	O157_vB-3r-2	O157_vB genome	9,944	KOD FX Neo	50	98	10	64	30	68	11	40
	4	O157_vB-4f-2	O157_vB-4r-2	O157_vB genome	10,379	KOD FX Neo	50	98	10	64	30	68	11	40
	5	O157_vB-5f-2	O157_vB-5r-2	O157_vB genome	10,187	KOD FX Neo	50	98	10	64	30	68	11	40
	6	O157_vB-6f-2	O157_vB-6r-2	O157_vB genome	9,252	KOD FX Neo	50	98	10	64	30	68	11	40
	7	O157_vB-7f-2	O157_vB-7r-2	O157_vB genome	8,802	KOD FX Neo	50	98	10	64	30	68	11	40
O157_vB$_{\mathrm{HiBiT(gp27)}}$	gp27-1	O157_vB-1f-2	HiBiT-gp27-r	O157_vB genome	4,450	KOD FX Neo	50	98	10	63	30	68	5	40
	gp27-2	HiBiT-gp27-f	O157_vB-1r-2	O157_vB genome	5,614	KOD FX Neo	50	98	10	63	30	68	8	40
	2	O157_vB-2f-2	O157_vB-2r-2	O157_vB genome	10,168	KOD FX Neo	50	98	10	64	30	68	11	40
	3	O157_vB-3f-2	O157_vB-3r-2	O157_vB genome	9,944	KOD FX Neo	50	98	10	64	30	68	11	40
	4	O157_vB-4f-2	O157_vB-4r-2	O157_vB genome	10,379	KOD FX Neo	50	98	10	64	30	68	11	40
	5	O157_vB-5f-2	O157_vB-5r-2	O157_vB genome	10,187	KOD FX Neo	50	98	10	64	30	68	11	40
	6	O157_vB-6f-2	O157_vB-6r-2	O157_vB genome	9,252	KOD FX Neo	50	98	10	64	30	68	11	40
	7	O157_vB-7f-2	O157_vB-7r-2	O157_vB genome	8,802	KOD FX Neo	50	98	10	64	30	68	11	40
O157_vB$_{\mathrm{HiBiT(gp32)}}$	gp32-1	O157_vB-1f-2	HiBiT-gp32-r	O157_vB genome	8,113	KOD FX Neo	50	98	10	64	30	68	11	40
	gp32-2	HiBiT-gp32-f	O157_vB-1r-2	O157_vB genome	1,920	KOD FX Neo	50	98	10	65	30	68	2	40
	2	O157_vB-2f-2	O157_vB-2r-2	O157_vB genome	10,168	KOD FX Neo	50	98	10	64	30	68	11	40
	3	O157_vB-3f-2	O157_vB-3r-2	O157_vB genome	9,944	KOD FX Neo	50	98	10	64	30	68	11	40
	4	O157_vB-4f-2	O157_vB-4r-2	O157_vB genome	10,379	KOD FX Neo	50	98	10	64	30	68	11	40
	5	O157_vB-5f-2	O157_vB-5r-2	O157_vB genome	10,187	KOD FX Neo	50	98	10	64	30	68	11	40
	6	O157_vB-6f-2	O157_vB-6r-2	O157_vB genome	9,252	KOD FX Neo	50	98	10	64	30	68	11	40
	7	O157_vB-7f-2	O157_vB-7r-2	O157_vB genome	8,802	KOD FX Neo	50	98	10	64	30	68	11	40
O157_vB$_{\mathrm{HiBiT(gp33)}}$	gp33-1	O157_vB-1f-2	HiBiT-gp33-r	O157_vB genome	9,916	KOD FX Neo	50	98	10	64	30	68	11	40
	gp33-2	HiBiT-gp33-f	O157_vB-2r-2	O157_vB genome	10,186	KOD FX Neo	50	98	10	64	30	68	11	40
	3	O157_vB-3f-2	O157_vB-3r-2	O157_vB genome	9,944	KOD FX Neo	50	98	10	64	30	68	11	40
	4	O157_vB-4f-2	O157_vB-4r-2	O157_vB genome	10,379	KOD FX Neo	50	98	10	64	30	68	11	40
	5	O157_vB-5f-2	O157_vB-5r-2	O157_vB genome	10,187	KOD FX Neo	50	98	10	64	30	68	11	40
	6	O157_vB-6f-2	O157_vB-6r-2	O157_vB genome	9,252	KOD FX Neo	50	98	10	64	30	68	11	40
	7	O157_vB-7f-2	O157_vB-7r-2	O157_vB genome	8,802	KOD FX Neo	50	98	10	64	30	68	11	40
O157_vB$_{\mathrm{HiBiT(gp35)}}$	gp35-1	O157_vB-1f-2	HiBiT-gp35-r	O157_vB genome	10,161	KOD FX Neo	50	98	10	64	30	68	11	40
	gp35-2	HiBiT-gp35-f	O157_vB-2r-2	O157_vB genome	9,941	KOD FX Neo	50	98	10	64	30	68	11	40
	3	O157_vB-3f-2	O157_vB-3r-2	O157_vB genome	9,944	KOD FX Neo	50	98	10	64	30	68	11	40
	4	O157_vB-4f-2	O157_vB-4r-2	O157_vB genome	10,379	KOD FX Neo	50	98	10	64	30	68	11	40
	5	O157_vB-5f-2	O157_vB-5r-2	O157_vB genome	10,187	KOD FX Neo	50	98	10	64	30	68	11	40
	6	O157_vB-6f-2	O157_vB-6r-2	O157_vB genome	9,252	KOD FX Neo	50	98	10	64	30	68	11	40
	7	O157_vB-7f-2	O157_vB-7r-2	O157_vB genome	8,802	KOD FX Neo	50	98	10	64	30	68	11	40
O157_vB$_{\mathrm{HiBiT(gp36)}}$	1	O157_vB-1f-2	O157_vB-1r-2	O157_vB genome	10,033	KOD FX Neo	50	98	10	64	30	68	11	40
	gp36-1	O157_vB-2f-2	HiBiT-gp36-r	O157_vB genome	3,176	KOD FX Neo	50	98	10	63	30	68	5	40
	gp36-2	HiBiT-gp36-f	O157_vB-2r-2	O157_vB genome	6,992	KOD FX Neo	50	98	10	63	30	68	8	40
	3	O157_vB-3f-2	O157_vB-3r-2	O157_vB genome	9,944	KOD FX Neo	50	98	10	64	30	68	11	40
	4	O157_vB-4f-2	O157_vB-4r-2	O157_vB genome	10,379	KOD FX Neo	50	98	10	64	30	68	11	40
	5	O157_vB-5f-2	O157_vB-5r-2	O157_vB genome	10,187	KOD FX Neo	50	98	10	64	30	68	11	40
	6	O157_vB-6f-2	O157_vB-6r-2	O157_vB genome	9,252	KOD FX Neo	50	98	10	64	30	68	11	40
	7	O157_vB-7f-2	O157_vB-7r-2	O157_vB genome	8,802	KOD FX Neo	50	98	10	64	30	68	11	40
O157_vB$_{\mathrm{HiBiT(gp37)}}$, Second	1	O157_vB-1f-2	O157_vB-1r-2	O157_vB genome	10,033	KOD FX Neo	50	98	10	64	30	68	11	40
	gp37-1	O157_vB-2f-2	HiBiT-gp37-r-2	O157_vB genome	4,029	KOD FX Neo	50	98	10	63	30	68	5	40

	gp37-2	HiBiT-gp37-f-2	O157_vB-2r-2	O157_vB genome	6,139	KOD FX Neo	50	98	10	63	30	68	8	40
	3	O157_vB-3f-2	O157_vB-3r-2	O157_vB genome	9,944	KOD FX Neo	50	98	10	64	30	68	11	40
	4	O157_vB-4f-2	O157_vB-4r-2	O157_vB genome	10,379	KOD FX Neo	50	98	10	64	30	68	11	40
	5	O157_vB-5f-2	O157_vB-5r-2	O157_vB genome	10,187	KOD FX Neo	50	98	10	64	30	68	11	40
	6	O157_vB-6f-2	O157_vB-6r-2	O157_vB genome	9,252	KOD FX Neo	50	98	10	64	30	68	11	40
	7	O157_vB-7f-2	O157_vB-7r-2	O157_vB genome	8,802	KOD FX Neo	50	98	10	64	30	68	11	40
O157_vB$_{\mathrm{HiBiT(gp41)}}$	1	O157_vB-1f-2	O157_vB-1r-2	O157_vB genome	10,033	KOD FX Neo	50	98	10	64	30	68	11	40
	gp41-1	O157_vB-2f-2	HiBiT-gp41-r	O157_vB genome	7,558	KOD FX Neo	50	98	10	63	30	68	8	40
	gp41-2	HiBiT-gp41-f	O157_vB-2r-2	O157_vB genome	2,610	KOD FX Neo	50	98	10	63	30	68	5	40
	3	O157_vB-3f-2	O157_vB-3r-2	O157_vB genome	9,944	KOD FX Neo	50	98	10	64	30	68	11	40
	4	O157_vB-4f-2	O157_vB-4r-2	O157_vB genome	10,379	KOD FX Neo	50	98	10	64	30	68	11	40
	5	O157_vB-5f-2	O157_vB-5r-2	O157_vB genome	10,187	KOD FX Neo	50	98	10	64	30	68	11	40
	6	O157_vB-6f-2	O157_vB-6r-2	O157_vB genome	9,252	KOD FX Neo	50	98	10	64	30	68	11	40
	7	O157_vB-7f-2	O157_vB-7r-2	O157_vB genome	8,802	KOD FX Neo	50	98	10	64	30	68	11	40
O157_vB$_{\mathrm{HiBiT(gp42)}}$	1	O157_vB-1f-2	O157_vB-1r-2	O157_vB genome	10,033	KOD FX Neo	50	98	10	64	30	68	11	40
	gp42-1	O157_vB-2f-2	HiBiT-gp42-r	O157_vB genome	8,712	KOD FX Neo	50	98	10	64	30	68	11	40
	gp42-2	HiBiT-gp42-f	O157_vB-2r-2	O157_vB genome	1,456	KOD FX Neo	50	98	10	65	30	68	2	40
	3	O157_vB-3f-2	O157_vB-3r-2	O157_vB genome	9,944	KOD FX Neo	50	98	10	64	30	68	11	40
	4	O157_vB-4f-2	O157_vB-4r-2	O157_vB genome	10,379	KOD FX Neo	50	98	10	64	30	68	11	40
	5	O157_vB-5f-2	O157_vB-5r-2	O157_vB genome	10,187	KOD FX Neo	50	98	10	64	30	68	11	40
	6	O157_vB-6f-2	O157_vB-6r-2	O157_vB genome	9,252	KOD FX Neo	50	98	10	64	30	68	11	40
	7	O157_vB-7f-2	O157_vB-7r-2	O157_vB genome	8,802	KOD FX Neo	50	98	10	64	30	68	11	40
O157_vB$_{\mathrm{HiBiT(gp48)}}$	1	O157_vB-1f-2	O157_vB-1r-2	O157_vB genome	10,033	KOD FX Neo	50	98	10	64	30	68	11	40
	2	O157_vB-2f-2	O157_vB-2r-2	O157_vB genome	10,168	KOD FX Neo	50	98	10	64	30	68	11	40
	gp48-1	O157_vB-3f-2	HiBiT-gp48-r	O157_vB genome	2,698	KOD FX Neo	50	98	10	63	30	68	5	40
	gp48-2	HiBiT-gp48-f	O157_vB-3r-2	O157_vB genome	7,246	KOD FX Neo	50	98	10	63	30	68	8	40
	4	O157_vB-4f-2	O157_vB-4r-2	O157_vB genome	10,379	KOD FX Neo	50	98	10	64	30	68	11	40
	5	O157_vB-5f-2	O157_vB-5r-2	O157_vB genome	10,187	KOD FX Neo	50	98	10	64	30	68	11	40
	6	O157_vB-6f-2	O157_vB-6r-2	O157_vB genome	9,252	KOD FX Neo	50	98	10	64	30	68	11	40
	7	O157_vB-7f-2	O157_vB-7r-2	O157_vB genome	8,802	KOD FX Neo	50	98	10	64	30	68	11	40
O157_vB$_{\mathrm{HiBiT(gp55)}}$	1	O157_vB-1f-2	O157_vB-1r-2	O157_vB genome	10,033	KOD FX Neo	50	98	10	64	30	68	11	40
	2	O157_vB-2f-2	O157_vB-2r-2	O157_vB genome	10,168	KOD FX Neo	50	98	10	64	30	68	11	40
	gp55-1	O157_vB-3f-2	HiBiT-gp55-r	O157_vB genome	5,754	KOD FX Neo	50	98	10	63	30	68	8	40
	gp55-2	HiBiT-gp55-f	O157_vB-3r-2	O157_vB genome	4,190	KOD FX Neo	50	98	10	63	30	68	5	40
	4	O157_vB-4f-2	O157_vB-4r-2	O157_vB genome	10,379	KOD FX Neo	50	98	10	64	30	68	11	40
	5	O157_vB-5f-2	O157_vB-5r-2	O157_vB genome	10,187	KOD FX Neo	50	98	10	64	30	68	11	40
	6	O157_vB-6f-2	O157_vB-6r-2	O157_vB genome	9,252	KOD FX Neo	50	98	10	64	30	68	11	40
	7	O157_vB-7f-2	O157_vB-7r-2	O157_vB genome	8,802	KOD FX Neo	50	98	10	64	30	68	11	40
O157_vB$_{\mathrm{HiBiT(gp57)}}$	1	O157_vB-1f-2	O157_vB-1r-2	O157_vB genome	10,033	KOD FX Neo	50	98	10	64	30	68	11	40
	2	O157_vB-2f-2	O157_vB-2r-2	O157_vB genome	10,168	KOD FX Neo	50	98	10	64	30	68	11	40
	gp57-1	O157_vB-3f-2	HiBiT-gp57-r	O157_vB genome	8,356	KOD FX Neo	50	98	10	64	30	68	11	40
	gp57-2	HiBiT-gp57-f	O157_vB-3r-2	O157_vB genome	1,589	KOD FX Neo	50	98	10	65	30	68	2	40
	4	O157_vB-4f-2	O157_vB-4r-2	O157_vB genome	10,379	KOD FX Neo	50	98	10	64	30	68	11	40
	5	O157_vB-5f-2	O157_vB-5r-2	O157_vB genome	10,187	KOD FX Neo	50	98	10	64	30	68	11	40
	6	O157_vB-6f-2	O157_vB-6r-2	O157_vB genome	9,252	KOD FX Neo	50	98	10	64	30	68	11	40
	7	O157_vB-7f-2	O157_vB-7r-2	O157_vB genome	8,802	KOD FX Neo	50	98	10	64	30	68	11	40
O157_vB$_{\mathrm{HiBiT(gp62)}}$	1	O157_vB-1f-2	O157_vB-1r-2	O157_vB genome	10,033	KOD FX Neo	50	98	10	64	30	68	11	40
	2	O157_vB-2f-2	O157_vB-2r-2	O157_vB genome	10,168	KOD FX Neo	50	98	10	64	30	68	11	40
	3	O157_vB-3f-2	O157_vB-3r-2	O157_vB genome	9,944	KOD FX Neo	50	98	10	64	30	68	11	40
	gp62-1	O157_vB-4f-2	HiBiT-gp62-r	O157_vB genome	4,706	KOD FX Neo	50	98	10	63	30	68	5	40
	gp62-2	HiBiT-gp62-f	O157_vB-4r-2	O157_vB genome	5,674	KOD FX Neo	50	98	10	63	30	68	8	40
	5	O157_vB-5f-2	O157_vB-5r-2	O157_vB genome	10,187	KOD FX Neo	50	98	10	64	30	68	11	40
	6	O157_vB-6f-2	O157_vB-6r-2	O157_vB genome	9,252	KOD FX Neo	50	98	10	64	30	68	11	40
	7	O157_vB-7f-2	O157_vB-7r-2	O157_vB genome	8,802	KOD FX Neo	50	98	10	64	30	68	11	40
O157_vB$_{\mathrm{HiBiT(gp64)}}$, Second	1	O157_vB-1f-2	O157_vB-1r-2	O157_vB genome	10,033	KOD FX Neo	50	98	10	64	30	68	11	40
	2	O157_vB-2f-2	O157_vB-2r-2	O157_vB genome	10,168	KOD FX Neo	50	98	10	64	30	68	11	40
	3	O157_vB-3f-2	O157_vB-3r-2	O157_vB genome	9,944	KOD FX Neo	50	98	10	64	30	68	11	40
	gp64-1	O157_vB-4f-2	HiBiT-gp64-r-2	O157_vB genome	7,157	KOD FX Neo	50	98	10	63	30	68	8	40
	gp64-2	HiBiT-gp64-f-2	O157_vB-4r-2	O157_vB genome	3,223	KOD FX Neo	50	98	10	63	30	68	5	40
	5	O157_vB-5f-2	O157_vB-5r-2	O157_vB genome	10,187	KOD FX Neo	50	98	10	64	30	68	11	40
	6	O157_vB-6f-2	O157_vB-6r-2	O157_vB genome	9,252	KOD FX Neo	50	98	10	64	30	68	11	40
	7	O157_vB-7f-2	O157_vB-7r-2	O157_vB genome	8,802	KOD FX Neo	50	98	10	64	30	68	11	40
O157_vB$_{\mathrm{HiBiT(gp69)}}$	1	O157_vB-1f-2	O157_vB-1r-2	O157_vB genome	10,033	KOD FX Neo	50	98	10	64	30	68	11	40
	2	O157_vB-2f-2	O157_vB-2r-2	O157_vB genome	10,168	KOD FX Neo	50	98	10	64	30	68	11	40
	3	O157_vB-3f-2	O157_vB-3r-2	O157_vB genome	9,944	KOD FX Neo	50	98	10	64	30	68	11	40
	gp69-1	O157_vB-4f-2	HiBiT-gp69-r	O157_vB genome	9,723	KOD FX Neo	50	98	10	64	30	68	11	40
	gp69-2	HiBiT-gp69-f	O157_vB-5r-2	O157_vB genome	10,752	KOD FX Neo	50	98	10	64	30	68	11	40
	6	O157_vB-6f-2	O157_vB-6r-2	O157_vB genome	9,252	KOD FX Neo	50	98	10	64	30	68	11	40
	7	O157_vB-7f-2	O157_vB-7r-2	O157_vB genome	8,802	KOD FX Neo	50	98	10	64	30	68	11	40
O157_vB$_{\mathrm{HiBiT(gp75)}}$	1	O157_vB-1f-2	O157_vB-1r-2	O157_vB genome	10,033	KOD FX Neo	50	98	10	64	30	68	11	40
	2	O157_vB-2f-2	O157_vB-2r-2	O157_vB genome	10,168	KOD FX Neo	50	98	10	64	30	68	11	40
	3	O157_vB-3f-2	O157_vB-3r-2	O157_vB genome	9,944	KOD FX Neo	50	98	10	64	30	68	11	40
	4	O157_vB-4f-2	O157_vB-4r-2	O157_vB genome	10,379	KOD FX Neo	50	98	10	64	30	68	11	40
	gp75-1	O157_vB-5f-2	HiBiT-gp75-r	O157_vB genome	3,749	KOD FX Neo	50	98	10	63	30	68	5	40
	gp75-2	HiBiT-gp75-f	O157_vB-5r-2	O157_vB genome	6,438	KOD FX Neo	50	98	10	63	30	68	8	40
	6	O157_vB-6f-2	O157_vB-6r-2	O157_vB genome	9,252	KOD FX Neo	50	98	10	64	30	68	11	40
	7	O157_vB-7f-2	O157_vB-7r-2	O157_vB genome	8,802	KOD FX Neo	50	98	10	64	30	68	11	40
O157_vB$_{\mathrm{HiBiT(gp85)}}$	1	O157_vB-1f-2	O157_vB-1r-2	O157_vB genome	10,033	KOD FX Neo	50	98	10	64	30	68	11	40
	2	O157_vB-2f-2	O157_vB-2r-2	O157_vB genome	10,168	KOD FX Neo	50	98	10	64	30	68	11	40
	3	O157_vB-3f-2	O157_vB-3r-2	O157_vB genome	9,944	KOD FX Neo	50	98	10	64	30	68	11	40
	4	O157_vB-4f-2	O157_vB-4r-2	O157_vB genome	10,379	KOD FX Neo	50	98	10	64	30	68	11	40
	5	O157_vB-5f-2	O157_vB-5r-2	O157_vB genome	10,187	KOD FX Neo	50	98	10	64	30	68	11	40
	gp85-1	O157_vB-6f-2	HiBiT-gp85-r	O157_vB genome	4,067	KOD FX Neo	50	98	10	63	30	68	5	40
	gp85-2	HiBiT-gp85-f	O157_vB-6r-2	O157_vB genome	5,185	KOD FX Neo	50	98	10	63	30	68	8	40
	7	O157_vB-7f-2	O157_vB-7r-2	O157_vB genome	8,802	KOD FX Neo	50	98	10	64	30	68	11	40
O157_vB$_{\mathrm{HiBiT(gp86)}}$	1	O157_vB-1f-2	O157_vB-1r-2	O157_vB genome	10,033	KOD FX Neo	50	98	10	64	30	68	11	40
	2	O157_vB-2f-2	O157_vB-2r-2	O157_vB genome	10,168	KOD FX Neo	50	98	10	64	30	68	11	40
	3	O157_vB-3f-2	O157_vB-3r-2	O157_vB genome	9,944	KOD FX Neo	50	98	10	64	30	68	11	40
	4	O157_vB-4f-2	O157_vB-4r-2	O157_vB genome	10,379	KOD FX Neo	50	98	10	64	30	68	11	40
	5	O157_vB-5f-2	O157_vB-5r-2	O157_vB genome	10,187	KOD FX Neo	50	98	10	64	30	68	11	40
	gp86-1	O157_vB-6f-2	HiBiT-gp86-r	O157_vB genome	5,631	KOD FX Neo	50	98	10	63	30	68	8	40
	gp86-2	HiBiT-gp86-f	O157_vB-6r-2	O157_vB genome	3,621	KOD FX Neo	50	98	10	63	30	68	5	40
	7	O157_vB-7f-2	O157_vB-7r-2	O157_vB genome	8,802	KOD FX Neo	50	98	10	64	30	68	11	40
O157_vB$_{\mathrm{HiBiT(gp91)}}$	1	O157_vB-1f-2	O157_vB-1r-2	O157_vB genome	10,033	KOD FX Neo	50	98	10	64	30	68	11	40
	2	O157_vB-2f-2	O157_vB-2r-2	O157_vB genome	10,168	KOD FX Neo	50	98	10	64	30	68	11	40
	3	O157_vB-3f-2	O157_vB-3r-2	O157_vB genome	9,944	KOD FX Neo	50	98	10	64	30	68	11	40
	4	O157_vB-4f-2	O157_vB-4r-2	O157_vB genome	10,379	KOD FX Neo	50	98	10	64	30	68	11	40
	5	O157_vB-5f-2	O157_vB-5r-2	O157_vB genome	10,187	KOD FX Neo	50	98	10	64	30	68	11	40
	gp91-1	O157_vB-6f-2	HiBiT-gp91-r	O157_vB genome	8,611	KOD FX Neo	50	98	10	64	30	68	11	40
	gp91-2	HiBiT-gp91-f	O157_vB-7r-2	O157_vB genome	9,343	KOD FX Neo	50	98	10	64	30	68	11	40

Supplementary Table 10: PCR conditions for amplification of HiBiT sequence.

Phage (PCR template)	Primer 1	Primer 2	Length (bp)	DNA polymerase	Reaction volume (μL)	Denature temperature (°C)	Denature time (s)	Annealing temperature (°C)	Annealing time (s)	Extension temperature (°C)	Extension time (min)	Cycles
$O157\_vB_{HiBiT(gp19)}$	HiBiT Check-gp19-f	HiBiT Check-gp19-r	472	Quick Taq HS DyeMix (Toyobo Co., Ltd., Osaka, Japan)	25	94	30	58	30	68	1	25
$O157\_vB_{HiBiT(gp20)}$	HiBiT Check-gp20-f	HiBiT Check-gp20-r	489	Quick Taq HS DyeMix	25	94	30	58	30	68	1	25
$O157\_vB_{HiBiT(gp27)}$	HiBiT Check-gp27-f	HiBiT Check-gp27-r	462	Quick Taq HS DyeMix	25	94	30	58	30	68	1	25
$O157\_vB_{HiBiT(gp32)}$	HiBiT Check-gp32-f	HiBiT Check-gp32-r	493	Quick Taq HS DyeMix	25	94	30	58	30	68	1	25
$O157\_vB_{HiBiT(gp35)}$	HiBiT Check-gp35-f	HiBiT Check-gp35-r	481	Quick Taq HS DyeMix	25	94	30	58	30	68	1	25
$O157\_vB_{HiBiT(gp36)}$	HiBiT Check-gp36-f	HiBiT Check-gp36-r	452	Quick Taq HS DyeMix	25	94	30	58	30	68	1	25
$O157\_vB_{HiBiT(gp37)}$, Second	HiBiT Check-gp37-f	HiBiT Check-gp37-r	449	Quick Taq HS DyeMix	25	94	30	58	30	68	1	25
$O157\_vB_{HiBiT(gp48)}$	HiBiT Check-gp48-f	HiBiT Check-gp48-r	495	Quick Taq HS DyeMix	25	94	30	58	30	68	1	25
$O157\_vB_{HiBiT(gp55)}$	HiBiT Check-gp55-f	HiBiT Check-gp55-r	490	Quick Taq HS DyeMix	25	94	30	58	30	68	1	25
$O157\_vB_{HiBiT(gp62)}$	HiBiT Check-gp62-f	HiBiT Check-gp62-r	467	Quick Taq HS DyeMix	25	94	30	58	30	68	1	25
$O157\_vB_{HiBiT(gp64)}$, Second	HiBiT Check-gp64-f	HiBiT Check-gp64-r	489	Quick Taq HS DyeMix	25	94	30	58	30	68	1	25
$O157\_vB_{HiBiT(gp75)}$	HiBiT Check-gp75-f	HiBiT Check-gp75-r	492	Quick Taq HS DyeMix	25	94	30	58	30	68	1	25
$O157\_vB_{HiBiT(gp85)}$	HiBiT Check-gp85-f	HiBiT Check-gp85-r	481	Quick Taq HS DyeMix	25	94	30	58	30	68	1	25
$O157\_vB_{HiBiT(gp86)}$	HiBiT Check-gp86-f	HiBiT Check-gp86-r	496	Quick Taq HS DyeMix	25	94	30	58	30	68	1	25
$O157\_vB_{HiBiT(gp91)}$	HiBiT Check-gp91-f	HiBiT Check-gp91-r	471	Quick Taq HS DyeMix	25	94	30	58	30	68	1	25

Supplementary Table 11: Whole genome re-sequencing of phage vB_Eco4M-7.

Locus tag	Product	Description
		vB_Eco4M-7 has a 68,194-bp genome with a 111-bp perfect direct repeat at both ends.
vBEco4M7_18	hypothetical protein	A→G, missense_variant Asp121Gly
vBEco4M7_38	hypothetical protein	AC→C, frameshift_variant Thr209fs
vBEco4M7_62	Putative structural protein	DNA sequence alignment: Identity 2451/2740 (89.45%), Gaps 2/2740 (0.07%)<br>Protein sequence alignment: Identity 878/913 (96.17%), Similarity 893/913 (97.81%), Gaps 0/913 (0.00%)<br><br>ATGCAAAAGTTTGACACAAACCTGTTGCAAAACCTTAAGTGGATGCAGAACAATGCACCAAACATTACATCTTTGGTGCAAAAGAAAAGTGACTGGTATGAAAGATACCA<br>AGATCAGTTTTGGACTGACTGGTACAATAACATTTTTAACATTGACACTTGTGATGCAATGGGCATTTATATTTGGTGCGACATTCTTTGCATACCAAGAGACTTTGTGGAC<br>TTAAGCAATTATGAGCACAACTGGGCGTTTGGACCGACGCGCGACAACTTTGAAGGTGCGGACAACATCGGCGGCAACTTCTTTGGTGCTGGCGGGAAGGTTGTAAAGAA<br>TATCGAAGAAGCGCGAATACTTCTTAAGCTTCGTTATCTGACGCTTACAAGTGATGGCCGCATCGAAAGTATCAACCGCATGCTTAAGTGGATTTTTAACCGTGGAGAGGC<br>TTGGGATTACTCTTCATCACGCTACGCATATGTGACCGACAATACTCAAGGTGCGATGTCTTCATCTGAATCAACAAATGGCTCATTCCTTCTGTACGACTGGCAAGGGTC<br>AACTGAGATTCAGCATGATTATAAGCAGATACTTTTAAGCCATGGCAACAACGCCGGGCTGACCATCAGCGGAGTGTCAGGCACCGTGTCACGGATGGCGCACCCAGATT<br>GGTACTCATGGGGAAATGGTTCCGGCACAGGGCTTGTCGGCGGAGACACCAGACAGTATGGTGGGCCTGATTACTATGGGCAGTTGTGTGGTGATTACCCAGGCGAGGCC<br>ATAGAGAATTTTGCCAACGCAGTAAGGGTCATCAAGACGGCTTCAAGCGGTGAGCCTGTGTATTCTTTGGCCAATAATCCAAGTGCCAAATCAATTTCGCTTCCTTCGCAG<br>GCTGGCGTCAACGTTTGCTATAGCTTCTACATAAAGGTGCTTTCTGAAAATGTCGCTTCTGTTTCTGTTACAGAGAATAGTGATGCATCGAGTAATGGAAAGCTGTTTGACC<br>CCGTCACTGGCCATTTCAACTTGGACCCGTCATTGAGCTATTTCATCAAAGGCAAATTCGATGGAGTAAGAAGGGAATCAAACCGTGGTGGCATAAAAGCAATTGGCAGC<br>GATTGGTATCGAATCTGGATTGTAGAGCAGACGGCAACCGCTGCTTCTGGAGTAAGTACCAGCTTTTCAATAGAGTTGAAGGCGACTGCCGCTGGATCAACTCAAACTCGT<br>GAATTGCCTGAAGGGTCTGATGCTCTTTTGTTTGGATACTCGATTTGCGTCTGCGACCCTGTTCAGAATCCAAATGGACCTGAGCCTTATTTTTATGGAACGCAAAACCCTC<br>CTTCTGACGGAACAGCAATCCTGTCTGTAACGGCGGGAACACTCAATAACATCTCCTGTGCAAACGGACCGCTGACGACAAGAAACGGTGTAGTTGGCGAGTCATTCATG<br>CAACCTGGCTCATCACTTGCATACTCTGGCTCAATCGGCGGCTACCCGTACCCAGGGAACGCCACGCTGATTGCAGAGACTGATGGAATCAGACGCTCATGGAACGTTAG<br>CAGGCTGGATGGAGTACCGCCGCCGCCGACCGCCGACTATTACATGGAGTACGTTATCGGACCTTATTTCGCCAACAACCTGTCAGACAACTTTAAGATGCTAGTTGGCG<br>ACAGGAATGAAGGATTCTTGCCAGCAAATGCCGGAATACGCTGGCAGCTTTACCAAGAAGTCGCAGACTTATCTGATATCGATTTGACTGTCAGCGACATAACAGACACG<br>CGCGTAGGATTTGAAACGGAAGGTGTTGCGTGGATTAACACTGACGGTATCATCAGAGTTGGCGGCATTAACGAGTGGCCGCTGAACAACTTCAACGGTGTGATACGCCA<br>GCTTGAATCTTTGAAAGGTGTAAACCAGGTCGTCGACGGCGCGCTTGTCAACGACTATTCAATCTCAAAAGCATCAAGAGATGACTTTGGTCGTGAAATACCACGCACGG<br>CAATCACTAACTATTGGCGCTACACACAGCCAAATGACGGTACATCTTTTGGCAATATAATTGAGGATGGCACAAACACTGTAACAGCGACGCCAATCAGCGGTGTGCCC<br>AACCAGTTCCCATCATTCAAGCTTTCCGCTACAATAGGGACTGTGCCAGCATCCGGTTATGCACGCGCTGTACTTGCTACTAAATACACTGGCACCCTGACTGGCCCTTTG<br>TCATTTATCGCAATAGTCAAAAACAACACGGTTAACGGTCAAAAACTTTTTGTAACTGTACCGGATGGGATGGGTGGCTATATCCCTAAATTCTACAACATAGGCGCAGGT<br>GATTTGCAACGTATCGAAATTGACCTTGCCGCTGAGATTGTTGAAGGCGCTACCTGGGATGGATCAACAGGCGGCATCAGCATCGGTTTGATATCAGGGAAAGACTTGCCG<br>ACCGAAGTTGGTACGACTCTTGAACTTGATATTTTAAGCTTGATGCTTGTGTCTGGTAGTACGATGAATGCGCTTGCAATTTCAACAAGCGAAGATTTCGCAAAGAAAACTG<br>CGACTTCTATATACATCAAAAACCCAGGATCACTTGCCACTGGAGTTCAATTGATTGCTTCTAATGGTGATGTTGTAGCAACAGTAAACTTTCCGAGAAGTGCTGCTGGTG<br>GTTATGTTTCAAGCATGAACATAACTGACTTAACTGGTGATTGGGCATCAACCCTTTTATCAAAAATTAAATACATCGTGTAG

Supplementary references

1 Li, L. *et al.* Chemoenzymatic synthesis of the bacterial polysaccharide repeating unit undecaprenyl pyrophosphate and its analogs. *Nat Protoc* **11**, 1280-1298 (2016). https://doi.org:10.1038/nprot.2016.067

2 Gao, Y. *et al.* Biochemical characterization of WbdN, a beta1,3-glucosyltransferase involved in O-antigen synthesis in enterohemorrhagic *Escherichia coli* O157. *Glycobiology* **22**, 1092-1102 (2012). https://doi.org:10.1093/glycob/cws081

3 Albermann, C. & Beuttler, H. Identification of the GDP-N-acetyl-d-perosamine producing enzymes from *Escherichia coli* O157:H7. *FEBS Lett* **582**, 479-484 (2008). https://doi.org:10.1016/j.febslet.2008.01.005

4 Bhunia, A. K. One day to one hour: how quickly can foodborne pathogens be detected? *Future Microbiol* **9**, 935-946 (2014). https://doi.org:10.2217/fmb.14.61

5 National Advisory Committee on Microbiological Criteria for Foods. Response to questions posed by the food safety and inspection service regarding determination of the most appropriate technologies for the food safety and inspection service to adopt in performing routine and baseline microbiological analyses. *J Food Prot* **73**, 1160-1200 (2010). https://doi.org:10.4315/0362-028x-73.6.1160

6 Hussain, W., Ullah, M. W., Farooq, U., Aziz, A. & Wang, S. Bacteriophage-based advanced bacterial detection: concept, mechanisms, and applications. *Biosens Bioelectron* **177**, 112973 (2021). https://doi.org:10.1016/j.bios.2021.112973

7 Foddai, A. C. G. & Grant, I. R. Methods for detection of viable foodborne pathogens: current state-of-art and future prospects. *Appl Microbiol Biotechnol* **104**, 4281-4288 (2020). https://doi.org:10.1007/s00253-020-10542-x

8 Emerson, J. B. *et al.* Schrodinger's microbes: tools for distinguishing the living from the dead in microbial ecosystems. *Microbiome* **5**, 86 (2017). https://doi.org:10.1186/s40168-017-0285-3

9 Li, B., Liu, H. & Wang, W. Multiplex real-time PCR assay for detection of *Escherichia coli* O157:H7 and screening for non-O157 Shiga toxin-producing *E. coli*. *BMC Microbiol* **17**, 215 (2017). https://doi.org:10.1186/s12866-017-1123-2

10 Santos, S. B. *et al.* Bacteriophage-receptor binding proteins for multiplex detection of *Staphylococcus* and *Enterococcus* in blood. *Biotechnol Bioeng* **117**, 3286-3298 (2020). https://doi.org:10.1002/bit.27489

11 Sillankorva, S., Neubauer, P. & Azeredo, J. *Pseudomonas fluorescens* biofilms subjected to phage phiIBB-PF7A. *BMC Biotechnol* **8**, 79 (2008). https://doi.org:10.1186/1472-6750-8-79

12 Mosier-Boss, P. A. *et al.* Use of fluorescently labeled phage in the detection and identification of bacterial species. *Appl Spectrosc* **57**, 1138-1144 (2003). https://doi.org:10.1366/00037020360696008

13 Low, H. Z. *et al.* Fast and easy phage-tagging and live/dead analysis for the rapid monitoring of bacteriophage infection. *Front Microbiol* **11**, 602444 (2020). https://doi.org:10.3389/fmicb.2020.602444

14 van der Merwe, R. G., van Helden, P. D., Warren, R. M., Sampson, S. L. & Gey van Pittius, N. C. Phage-based detection of bacterial pathogens. *Analyst* **139**, 2617-2626 (2014). https://doi.org:10.1039/c4an00208c

15 Mahler, M., Costa, A. R., van Beljouw, S. P. B., Fineran, P. C. & Brouns, S. J. J. Approaches for bacteriophage genome engineering. *Trends Biotechnol* **41**, 669-685 (2023). https://doi.org:10.1016/j.tibtech.2022.08.008

16 Kim, J., Kim, M., Kim, S. & Ryu, S. Sensitive detection of viable *Escherichia coli* O157:H7 from foods using a luciferase-reporter phage phiV10lux. *Int J Food Microbiol* **254**, 11-17 (2017). https://doi.org:10.1016/j.ijfoodmicro.2017.05.002

17 Zhang, D. *et al.* The use of a novel NanoLuc-based reporter phage for the detection of *Escherichia coli* O157:H7. *Sci Rep* **6**, 33235 (2016). https://doi.org:10.1038/srep33235

18 Brigati, J. R. *et al.* Bacteriophage-based bioluminescent bioreporter for the detection of *Escherichia coli* 0157:H7. *J Food Prot* **70**, 1386-1392 (2007). https://doi.org:10.4315/0362-028x-70.6.1386

19 Azam, A. H. *et al.* Selective bacteriophages reduce the emergence of resistant bacteria in the bacteriophage-antibiotic combination therapy. *bioRxiv*, 2023.2001.2022.525106 (2023). https://doi.org:10.1101/2023.01.22.525106

20 Morita, M. *et al.* Characterization of a virulent bacteriophage specific for *Escherichia coli* O157:H7 and analysis of its cellular receptor and two tail fiber genes. *FEMS Microbiol Lett* **211**, 77-83 (2002). https://doi.org:10.1111/j.1574-6968.2002.tb11206.x

21 Necel, A. *et al.* Characterization of a bacteriophage, vB_Eco4M-7, that effectively infects many *Escherichia coli* O157 strains. *Sci Rep* **10**, 3743 (2020). https://doi.org:10.1038/s41598-020-60568-4

22 Iguchi, A., Iyoda, S., Watanabe, H. & Osawa, R. O side chain deficiency enhances sensitivity of *Escherichia coli* to Shiga toxin 2-converting bacteriophages. *Curr Microbiol* **54**, 14-19 (2007). https://doi.org:10.1007/s00284-006-0139-x
